# Supplementary material for: Factors associated with elder abuse and neglect in rural Uganda: A cross-sectional study of community older adults attending an outpatient clinic
Source: PLoS One. 2023 Feb 10;18(2):e0280826. doi: 10.1371/journal.pone.0280826 (PMC9916607; doi:10.1371/journal.pone.0280826)
Supplement: S1 Table — (DOCX) [file pone.0280826.s002.docx]

**Supplementary File**

Elder abuse Questionnaire

| **Question** | **Response** | |
| --- | --- | --- |
|  | Yes | No |
| **Emotional mistreatment is assessed by the following questions: (from the past 6 months)** | | |
| 1. “Has anyone ever verbally attacked, scolded, or yelled at you so that you felt afraid for your safety, threatened or intimidated?” |  |  |
| 1. “Has anyone ever made you feel humiliated or embarrassed by calling you names such as stupid, or telling you that you or your opinion was worthless? |  |  |
| 1. “Has anyone ever forcefully or repeatedly asked you to do some-thing so much that you felt harassed or coerced into doing something against your will?” |  |  |
| 1. “Has anyone close to you ever completely refused to talk to you or ignored you for days at a time, even when you wanted to talk to them? |  |  |
| **Physical mistreatment is defined as an affirmative answer (answer since the past 6 months) to any one of the following:** | | |
| 1. “Has anyone ever hit you with their hand or object, slapped you, or threatened you with a weapon?” |  |  |
| 1. “Has anyone ever tried to restrain you by holding you down, tying you up, or locking you in your room or house?” |  |  |
| 1. “Has anyone ever physically hurt you so that you suffered some degree of injury, including cuts, bruises, or other marks?” |  |  |
| **Sexual mistreatment is defined as an affirmative answer to any one of the following three question (answer since the past 6 months)** | | |
| 1. “Regardless of how long ago it happened or who made the advances, has anyone ever made you have sex or oral sex by using force or threatening to harm you or someone close to you?” |  |  |
| 1. For females - “Has anyone ever touched your breasts or pubic area or made you touch his penis by using force or threat of force?” |  |  |
| 1. For males - “Has anyone ever touched your pubic area or made you touch their pubic area by using force or threat of force?” |  |  |
| 1. For females - “Has anyone ever forced you to undress or expose your breasts or pubic area when you didn’t want to?” |  |  |
| 1. For males - “Has anyone ever forced you to undress or expose your pubic area when you didn’t want to?” |  |  |
| **Neglect in the past 6 months**  Neglect is defined as instances in which an older adult identified that they had one of the needs listed below, but this need was not always met.  “Now we would like to ask you some additional questions about whether there is someone who helps you with day-to-day things. You may not need help with any of these things, and if that is the case, just feel free to tell us you don’t need this type of help. Some older adults do need help with these things, so it’s important for us to ask (answer since the past 6 months) | | |
| 1. Do you need someone to help you get to the places you need to go, for example, do you need someone to drive you to the grocery store, a place of worship, the doctor?” |  |  |
| 1. “Do you need someone to make sure you have enough food, medicines, or any other things you need in your house?” |  |  |
| 1. “Do you need someone to help you with household things, like cooking meals, helping you eat, or making sure you take the correct medicines each day?” |  |  |
| 1. “Do you need someone to help you with house cleaning or yard work?” |  |  |
| 1. “Do you need someone to help you get out of bed, get showered, or get dressed?” |  |  |
| 1. “Do you need someone to make sure your bills get paid?” |  |  |
| **Financial Exploitation perpetrated by family members was determined by an affirmative answer to one of the following questions (answer since the past 6 months)**  Now we would like to ask your opinion about how your finances and property are handled | | |
| 1. “Is there someone who helps you take care of your finances, or is there someone other than yourself who makes decisions about your money and your property, either with or with-out your approval?” |  |  |
| 1. “Does that person ask for your PERMISSION before deciding to spend your money or sell your property?” |  |  |
| 1. “Do you feel like that person makes good decisions about your finances?” |  |  |
| 1. “Do you have the copies of paperwork for the financial decisions they make or can you get copies if you wanted them?” |  |  |
| 1. “Has that person ever forged your signature without your permission to sell your property or to get money from your accounts?” |  |  |
| 1. “Has that person ever forced or tricked you into signing a document so that they would be able to get some of your money or possessions? |  |  |
