# Supplementary material for: Factors associated with elder abuse and neglect in rural Uganda: A cross-sectional study of community older adults attending an outpatient clinic
Source: PLoS One. 2023 Feb 10;18(2):e0280826. doi: 10.1371/journal.pone.0280826 (PMC9916607; doi:10.1371/journal.pone.0280826)
Supplement: S2 Table — (DOCX) [file pone.0280826.s003.docx]

**Supplementary file 2. Relationship between the type of abuse and study variable**

| **Variable** | **Neglect** | | | **Financial abuse** | | | **Emotional mistreatment** | | | **Physical mistreatment** | | | **Sexual abuse** | | |
| --- | --- | --- | --- | --- | --- | --- | --- | --- | --- | --- | --- | --- | --- | --- | --- |
|  | No  n = 50 (13.7) | Yes  n = 351 (86.3) | *χ*^2^ (p-value) | No  n =194 (53.2) | Yes  n = 171 (46.8) | *χ*^2^ (p-value) | No  n = 186 (51.0) | Yes  n = 179 (49.0) | *χ*^2^ (p-value) | No  n = 284 (77.8) | Yes  n = 81 22.2) | *χ*^2^ (p-value) | No  n =340 (93.2) | Yes  n = 30 (6.8) | *χ*^2^ (p-value) |
| Age | | | | | | | | | | | | | | | |
| 60 – 69 | 40 (15.9) | 212 (84.1) | 5.17 (0.075) | 143 (56.8) | 109 (43.2) | 19.6 (<0.001) | 131 (52.0) | 121 (48.0) | 2.57 (0.276) | 195 (77.4) | 57 (22.6) | 8.36 (0.015) | 233 (92.5) | 19 (7.5) | 0.65 (0.723) |
| 70 – 79 | 10 (11.1) | 80 (88.9) |  | 49 (54.4) | 41 (45.6) |  | 47 (52.2) | 43 (47.8) |  | 76 (84.4) | 14 (15.6) |  | 85 (94.4) | 5 (5.6) |  |
| Above 80 | 0 | 23 (100) |  | 2 (8.7) | 21 (91.3) |  | 8 (34.8) | 15 (65.2) |  | 13 (56.5) | 10 (43.5) |  | 22 (95.6) | 1 (4.4) |  |
| Gender | | | | | | | | | | | | | | | |
| Female | 29 (13.9) | 179 (86.1) | 0.02 (0.876) | 123 (59.1) | 85 (40.9) | 6.95 (0.008) | 97 (46.6) | 111 (53.4) | 3.62 (0.057) | 162 (77.9) | 46 (22.1) | 0 (0.968) | 192 (92.3) | 16 (7.7) | 0.54 (0.463) |
| Male | 21 (13.3) | 136 (86.6) |  | 71 (45.2) | 86 (54.8) |  | 89 (56.7) | 68 (43.3) |  | 122 (77.7) | 35 (22.3) |  | 148 (94.3) | 9 (5.7) |  |
| Area of dwelling | | | | | | | | | | | | | | | |
| Rural | 39 (15.1) | 220 (84.9) | 1.39 (0.238) | 147 (56.8) | 112 (43.2) | 4.66 (0.031) | 131 (50.6) | 128 (49.4) | 0.05 (0.821) | 205 (79.1) | 54 (20.9) | 0.93 (0.335) | 240 (92.7) | 19 (7.3) | 0.33 (0.565) |
| Urban | 11 (10.4) | 95 (89.6) |  | 47 (44.3) | 59 (55.7) |  | 55 (51.9) | 51 (48.1) |  | 79 (74.5) | 27 (25.5) |  | 100 (94.3) | 6 (5.7) |  |
| Marital status | | | | | | | | | | | | | | | |
| Cohabiting or married | 25 (11.5) | 192 (88.5) | 2.86 (0.227) | 112 (51.6) | 105 (48.4) | 1.13 (0.771) | 115 (53.0) | 102 (47.0) | 5.20 (0.158) | 170 (78.3) | 47 (21.7) | 10.67 (0.014) | 200 (92.2) | 17 (7.8) | 0.97 (0.808) |
| Divorced or separated | 12 (20.7) | 46 (79.3) |  | 34 (58.6) | 24 (41.4) |  | 32 (55.2) | 26 (44.8) |  | 45 (77.6) | 13 (22.4) |  | 55 (94.8) | 3 (5.2) |  |
| Never married | 0 | 3 (100) |  | 2 (66.7) | 1 (33.3) |  | 0 | 3 (100) |  | 0 | 3 (100) |  | 3 (100) | 0 |  |
| Widowed | 13 (14.9) | 74 (85.1) |  | 46 (52.9) | 41 (47.1) |  | 39 (44.8) | 48 (55.2) |  | 69 (79.3) | 18 (20.7) |  | 82 (94.3) | 5 (5.7) |  |
| Employment status | | | | | | | | | | | | | | | |
| Previously formally employed, retired but currently still active | 3 (12.5) | 21 (87.5) | 3.51 (0.319) | 10 (41.7) | 14 (58.3) | 12.70 (0.005) | 11 (45.8) | 13 (54.2) | 9.08 (0.028) | 19 (79.2) | 5 (20.8) | 1.84 (0.605) | 20 (83.3) | 4 (16.7) | 5.04 (0.169) |
| Previously formally employed, retired but currently not active | 6 (14.6) | 35 (85.4) |  | 15 (36.6) | 26 (63.4) |  | 23 (56.1) | 18 (43.9) |  | 32 (78.0) | 9 (22.0) |  | 38 (92.7) | 3 (7.3) |  |
| Previously informally employed, currently not active | 2 (4.7) | 41 (95.4) |  | 17 (39.5) | 26 (60.5) |  | 13 (30.2) | 30 (69.8) |  | 30 (69.8) | 54 (30.2) |  | 42 (97.7) | 1 (2.3) |  |
| Previously informally employed and currently still active | 39 (15.2) | 218 (84.8) |  | 152 (59.1) | 105 (40.9) |  | 139 (54.1) | 118 (45.9) |  | 203 (79.0) | 54 (21.0) |  | 240 (93.4) | 17 (6.6) |  |
| Level of education | | | | | | | | | | | | | | | |
| Never | 7 (8.1) | 79 (91.9) | 3.84 (0.279) | 56 (65.1) | 30 (34.8) | 17.51 (0.001) | 48 (55.8) | 38 (44.2) | 3.06 (0.383) | 73 (84.9) | 13 (15.1) | 3.95 (0.266) | 83 (96.5) | 3 (3.5) | 2.24 (0.534) |
| Primary | 23 (13.9) | 142 (86.1) |  | 82 (49.7) | 83 (50.3) |  | 76 (46.1) | 89 (53.9) |  | 122 (73.9) | 43 (26.1) |  | 153 (92.7) | 12 (7.3) |  |
| Secondary | 13 (18.6) | 57.2 (81.4) |  | 43 (61.4) | 27 (38.6) |  | 39 (55.7) | 31 (44.3) |  | 55 (78.6) | 15 (21.4) |  | 64 (91.4) | 6 (8.6) |  |
| Tertiary | 7 (15.9) | 37 (84.1) |  | 13 (29.6) | 31 (70.4) |  | 23 (52.3) | 21 (47.7) |  | 34 (77.3) | 10 (22.7) |  | 40 (90.9) | 4 (9.1) |  |
| Type of housing | | | | | | | | | | | | | | | |
| Private | 42 (12.9) | 283 (87.1) | 4.36 (0.113) | 169 (52.0) | 156 (48.0) | 3.25 (0.197) | 166 (51.1) | 159 (48.9) | 3.77 (0.152) | 257 (79.1) | 68 (20.9) | 3.63 (0.163) | 301 (92.6) | 24 (7.4) | 1.59 (0.451) |
| Public | 5 (31.3) | 11 (68.7) |  | 12 (75.0) | 4 (25.0) |  | 11 (68.7) | 5 (31.2) |  | 12 (75.0) | 4 (25.0) |  | 16 (100) | 0 |  |
| Rental | 3 (12.5) | 21 (87.5) |  | 13 (54.2) | 11 (45.8) |  | 9 (37.5) | 15 (62.5) |  | 15 (62.5) | 9 (37.5) |  | 23 (95.8) | 1 (4.2) |  |
| Presence of a chronic illness | | | | | | | | | | | | | | | |
| No | 12 (11.9) | 89 (88.1) | 0.39 (0.532) | 43 (42.6) | 58 (57.4) | 6.27 (0.012) | 43 (42.6) | 58 (57.4) | 3.93 (0.047) | 66 (65.4) | 35 (34.6) | 12.6 (<0.001) | 96 (95.1) | 5 (4.9) | 0.79 (0.374) |
| Yes | 38 (14.4) | 226 (85.6) |  | 151 (57.2) | 113 (42.8) |  | 143 (54.2) | 121 (45.8) |  | 218 (82.6) | 46 (17.4) |  | 244 (92.4) | 20 (7.6) |  |
| Physical impairment | | | | | | | | | | | | | | | |
| No | 16 (7.9) | 187 (92.1) | 13.09 (<0.001) | 84 (41.4) | 119 (58.6) | 25.45 (<0.001) | 100 (49.3) | 103 (50.7) | 0.53 (0.468) | 153 (75.4) | 50 (24.6) | 1.57 (0.209) | 188 (92.6) | 15 (7.4) | 0.21 (0.648) |
| Yes | 34 (21.0) | 128 (79.0) |  | 110 (67.9) | 52 (32.1) |  | 86 (53.1) | 76 (46.9) |  | 131 (80.9) | 31 (19.1) |  | 152 (93.8) | 10 (6.2) |  |
| Reported perpetrators | | | | | | | | | | | | | | | |
| No | 47 (15.7) | 252 (84.3) | 5.71 (0.017) | 164 (54.8) | 135 (45.2) | 1.92 (0.166) | 177 (59.2) | 122 (40.8) | 44.91 (<0.001) | 251 (84.0) | 48 (16.0) | 26.08 (<0.001) | 279 (93.3) | 20 (6.7) | 0.07 (0.796) |
| Yes | 3 (4.6) | 63 (95.4) |  | 30 (45.4) | 36 (54.6) |  | 9 (13.6) | 57 (86.4) |  | 33 (50.0) | 33 (50.0) |  | 61 (92.4) | 5 (7.6) |  |
| History of reporting abuse to police | | | | | | | | | | | | | | | |
| No | 46 (13.9) | 286 (86.1) | 0.08 (0.782) | 169 (50.9) | 163 (49.1) | 7.45 (0.006) | 180 (54.2) | 152 (45.8) | 15.60 (<0.001) | 263 (79.2) | 69 (20.8) | 4.22 (0.040) | 310 (93.4) | 22 (6.6) | 0.29 (0.593) |
| Yes | 4 (12.1) | 29 (87.9) |  | 25 (75.8) | 8 (24.2) |  | 6 (18.2) | 27 (81.8) |  | 21 (63.6) | 12 (36.4) |  | 30 (90.9) | 3 (9.1) |  |
| Functional dependence | | | | | | | | | | | | | | | |
| severe dependency | 0 | 11 (100) | 18.78 (<0.001) | 2 (18.2) | 9 (81.8) | 8.63 (0.035) | 7 (63.6) | 4 (36.4) | 9.35 (0.025) | 10 (90.9) | 1 (9.1) | 13.05 (0.005) | 10 (90.9) | 1 (9.1) | 0.37 (0.946) |
| moderate dependency | 2 (2.9) | 67 (97.1) |  | 31 (44.9) | 38 (55.1) |  | 24 (34.8) | 45 (65.2) |  | 43 (62.3) | 26 (37.7) |  | 64 (92.7) | 5 (7.3) |  |
| slight dependency | 2 (4.2) | 46 (95.8) |  | 28 (58.3) | 20 (41.7) |  | 27 (56.2) | 21 (43.8) |  | 41 (85.4) | 7 (13.6) |  | 44 (91.7) | 4 (8.3) |  |
| Independent | 46 (19.4) | 191 (80.6) |  | 133 (56.1) | 104 (43.9) |  | 128 (54.0) | 109 (46.0) |  | 190 (80.2) | 47 (19.8) |  | 222 (93.7) | 15 (6.3) |  |
