# Supplementary material for: Factors associated with elder abuse and neglect in rural Uganda: A cross-sectional study of community older adults attending an outpatient clinic
Source: PLoS One. 2023 Feb 10;18(2):e0280826. doi: 10.1371/journal.pone.0280826 (PMC9916607; doi:10.1371/journal.pone.0280826)
Supplement: S3 Table — (DOCX) [file pone.0280826.s004.docx]

**Supplemntary file 3: Bivariate Logistic regression analysis for factors associated with the different types of abuse**

| **Variables** | **Neglect** | | **Financial abuse** | | **Emotional mistreatment** | | **Physical mistreatment** | | **Sexual abuse** | |
| --- | --- | --- | --- | --- | --- | --- | --- | --- | --- | --- |
|  | cOR (95% CI) | p-value | cOR (95% CI) | p-value | cOR (95% CI) | p-value | cOR (95% CI) | p-value | cOR (95% CI) | p-value |
| Age | | | | | | | | | | |
| 60 – 69 | 1 |  | 1 |  | 1 |  | 1 |  | 1 |  |
| 70 – 79 | 1.51 (0.72 – 3.16) | 0.275 | 1.10 (0.68 – 1.78 ) | 0.706 | 0.99 (0.61 – 1.60) | 0.969 | 0.63 (0.33 – 1.19) | 0.159 | 0.72 (0.26 – 1.99) | 0.529 |
| Above 80 | Omitted |  | 13.77 (3.16 – 60.01) | <0.001 | 2.02 (-.83 – 4.95) | 0.120 | 2.63 (1.09 – 6.32) | 0.030 | 0.56 (0.07 – 4.36) | 0.578 |
| Gender | | | | | | | | | | |
| Female | 1 |  | 1 |  | 1 |  | 1 |  | 1 |  |
| Male | 1.05 (0.57 – 1.92) | 0.876 | 1.75 (1.15 – 2.66) | 0.009 | 0.67 (0.44 – 1.01) | 0.058 | 1.01 (0.61 – 1.66) | 0.968 | 0.73 (0.31 – 1.70) | 0.465 |
| Area of dwelling | | | | | | | | | | |
| Rural | 1 |  | 1 |  | 1 |  | 1 |  | 1 |  |
| Urban | 1.53 (0.75 – 3.11) | 0.240 | 1.65 (1.04 – 2.60) | 0.032 | 0.95 (0.60 – 1.49) | 0.821 | 1.29 (0.76 – 2.20) | 0.335 | 0.76 (0.29 – 1.95) | 0.566 |
| Marital status | | | | | | | | | | |
| Cohabiting or married | 1 |  | 1 |  | 1 |  | 1 |  | 1 |  |
| Divorced or separated | 0.50 (0.23 – 1.06) | 0.073 | 0.75 (0.42 – 1.35) | 0.343 | 0.92 (0.51 – 1.64) | 0.768 | 1.04 (0.52 – 2.09) | 0.902 | 0.64 (0.18 – 2.27) | 0.491 |
| Never married | 0mitted |  | 0.53 (0.05 – 5.97) | 0.610 | Omitted |  | Omitted |  | Omitted |  |
| Widowed | 0.74 (0.36 – 1.52) | 0.416 | 0.95 (0.58 – 1.56) | 0.842 | 1.39 (0.84 – 2.29) | 0.199 | 0.94 (0.51 – 1.74) | 0.852 | 0.72 (0.26 – 2.01) | 0.527 |
| Employment status | | | | | | | | | | |
| Previously formally employed, retired but currently still active | 1 |  | 1 |  | 1 |  | 1 |  | 1 |  |
| Previously formally employed, retired but currently not active | 0.83 (0.19) | 0.810 | 1.24 (0.44 – 3.47) | 0.685 | 0.66 (0.24 – 1.82) | 0.425 | 1.07 (0.31 – 3.66) | 0.916 | 0.39 (0.08 – 1.94) | 0.252 |
| Previously informally employed, currently not active | 2.93 (0.45 – 18.90) | 0.259 | 1.09 (0.39 – 3.02) | 0.865 | 1.95 (0.69 – 5.49) | 0.204 | 1.65 (0.51 – 5.36) | 0.408 | 0.12 (0.01 – 1.13) | 0.064 |
| Previously informally employed and currently still active | 0.80 (0.23 – 2.81) | 0.726 | 0.49 (0.21 – 1.15) | 0.103 | 0.72 (0.31 – 1.66) | 0.440 | 1.02 (0.36 – 2.83) | 0.984 | 0.35 (0.11 – 1/15) | 0.085 |
| Level of education | | | | | | | | | | |
| Never | 1 |  | 1 |  | 1 |  | 1 |  | 1 |  |
| Primary | 0.55 (0.22 – 1.33) | 0.184 | 1.89 (1.10 – 3.24) | 0.021 | 1.48 (0.87 – 2.50) | 0.143 | 1.98 (0.99 – 3.92) | 0.051 | 2.17 (0.59 – 7.90) | 0.240 |
| Secondary | 0.39 (0.14 – 1.03) | 0.059 | 1.17 (0.61 – 2.25) | 0.634 | 1.00 (0.53 – 1.89) | 0.990 | 1.53 (0.67 – 3.48) | 0.309 | 2.59 (0.62 – 10.77) | 0.189 |
| Tertiary | 0.47 (0.15 – 1.43) | 0.184 | 4.45 (2.03 – 9.76) | <0.001 | 1.15 (0.56 – 2.39) | 0.701 | 1.65 (0.66 – 4.14) | 0.285 | 2.77 (0.59 – 12.95) | 0.196 |
| Type of housing | | | | | | | | | | |
| Private | 1 |  | 1 |  | 1 |  | 1 |  | 1 |  |
| Public | 0.33 (0.11 – 0.99) | 0.047 | 0.36 (0.11 – 1.14) | 0.083 | 0.47 (0.16 – 1.40) | 0.176 | 1.26 (0.39 – 4.03) | 0.697 | Omitted |  |
| Rental | 1.04 (0.27 – 3.63) | 0.952 | 0.92 (0.40 – 2.10) | 0.838 | 1.74 (0.74 – 4.09) | 0.204 | 2.27 (0.95 – 5.40) | 0.065 | 0.54 (0.07 – 4.21) | 0.561 |
| Presence of a chronic illness | | | | | | | | | | |
| No | 1 |  | 1 |  | 1 |  | 1 |  | 1 |  |
| Yes | 0.80 (0.40 – 1.60) | 0.533 | 0.55 (0.35 – 0.88) | 0.013 | 0.63 (0.39 – 0.99) | 0.048 | 0.40 (0.24 – 0.67) | <0.001 | 1.57 (0.57 – 4.31) | 0.378 |
| Physical impairment | | | | | | | | | | |
| No | 1 |  | 1 |  | 1 |  | 1 |  | 1 |  |
| Yes | 0.32 (0.17 – 0.61) | <0.001 | 0.33 (0.22 – 0.51) | <0.001 | 0.86 (0.57 – 1.30) | 0.468 | 0.72 (0.44 – 1.20) | 0.210 | 0.82 (0.36 – 1.89) | 0.648 |
| Reported perpetrators | | | | | | | | | | |
| No | 1 |  | 1 |  | 1 |  | 1 |  | 1 |  |
| Yes | 3.92 (1.18 – 12.99) | 0.026 | 1.46 (0.85 – 2.49) | 0.168 | 9.19 (4.38 – 19.26) | <0.001 | 5.23 (2.95 – 9.27) | <0.001 | 1.14 (0.41 – 3.17) | 0.796 |
| History of reporting abuse to police | | | | | | | | | | |
| No | 1 |  | 1 |  | 1 |  | 1 |  | 1 |  |
| Yes | 1.17 (0.39 – 3.47) | 0.782 | 0.33 (0.14 – 0.76) | 0.009 | 5.33 (2.14 – 13.25) | <0.001 | 2.18 (1.02 – 4.64) | 0.044 | 1.41 (0.40 – 4.98) | 0.595 |
| Functional dependence | | | | | | | | | | |
| Severe dependency | Omitted |  | 5.75 (1.22 – 27.21) | 0.027 | 0.67 (0.19 – 2.35) | 0.533 | 0.40 (0.05 – 3.24) | 0.393 | 1.48 (0.18 – 12.34) | 0.717 |
| Moderate dependency | 8.07 (1.91 – 34.15) | 0.005 | 1.57 (0.91 – 2.69) | 0.102 | 2.20 (1.26 – 3.84) | 0.006 | 2.44 (1.36 – 4.37) | 0.003 | 1.56 (0.40 – 3.30) | 0.786 |
| Slight dependency | 5.54 (1.20 – 23.66) | 0.021 | 0.91 (0.49 – 1.71) | 0.778 | 0.91 (0.49 – 1.71) | 0.776 | 0.69 (0.29 – 1.64) | 0.400 | 1.34 (0.42 – 4.25) | 0.613 |
| Independent | 1 |  | 1 |  | 1 |  | 1 |  | 1 |  |
