# Supplementary material for: Factors associated with elder abuse and neglect in rural Uganda: A cross-sectional study of community older adults attending an outpatient clinic
Source: PLoS One. 2023 Feb 10;18(2):e0280826. doi: 10.1371/journal.pone.0280826 (PMC9916607; doi:10.1371/journal.pone.0280826)
Supplement: Attachment — Submitted filename: PONE-D-22-24400_reviewer.pdf [file pone.0280826.s005.pdf]

PLOS ONE

Factors associated with elder abuse in rural Uganda: A hospital-based cross-sectional study

--Manuscript Draft--

**Manuscript Number:**	PONE-D-22-24400
**Article Type:**	Research Article
**Full Title:**	Factors associated with elder abuse in rural Uganda: A hospital-based cross-sectional study
**Short Title:**	Elderly abuse in Uganda
**Corresponding Author:**	Mark Mohan Kaggwa, M.D<br>Mbarara University of Science and Technology<br>Mbarara, UGANDA
**Keywords:**	Elderly abuse severity, types of elderly abuse, Hwalek- Sengstock elder abuse screening test, functional dependency, geriatrics, neglect, mistreatment, sexual abuse, emotional abuse, physical abuse, financial abuse, and Uganda
**Abstract:**	Background: There is a trend of a growing global population of older persons, especially in low-middle-income countries, and many older adults are vulnerable to abuse due to many factors, such as functional decline, increased elderly poverty, and neurocognitive disease, among others. Available literature shows that one in ten older persons is experiencing abuse. Abuse can impact the well-being of the elderly, decreasing their quality of life and leading to mental health challenges, morbidity, and mortality. Evidence on elderly abuse is vital to facilitate initiatives, but there are fewer studies on older adult abuse in Africa, particularly in Uganda. Objective: This study aimed to determine the prevalence of elderly abuse, the various types of elderly abuse and their associated factors in a Ugandan elderly sample. Methods: This was a hospital-based cross-sectional study among patients aged 60 years and above. Information on sociodemographic characteristics, functional impairment using the Barthel Index Scale, and elderly abuse severity using the Hwalek-Sengstock Elder Abuse Screening Test were collected. In addition, types of abuse, including neglect, physical, sexual, emotional, and financial abuse, were assessed using questions adapted from the US National Research Council on elder mistreatment monograph. Linear and logistic regression analyses were used to determine the factors associated with elderly abuse severity and the different types of abuse, respectively. Results: Overall, the prevalence of elder abuse was 89.0%. Neglect was the most common type of elderly abuse (86%), followed by emotional (49%), financial (46.8%), physical mistreatment (25%), and sexual (6.8%). About 30.4% of the abused elderlies experienced at least two forms of abuse. The factors associated with an increase in elderly abuse severity were having a secondary level of education and physical impairment. Moderate to severe functional dependence was associated with almost all forms of abuse. Individuals who reported the presence of a perpetrator were likely to experience neglect, emotional, and physical mistreatment. However, those who reported their perpetrators to the police had a higher likelihood of experiencing emotional abuse but were less likely to experience financial abuse. Emotional abuse was also associated with age above 80 years and attaining education (primary and secondary). Physical impairment and chronic medical conditions reduced the likelihood of experiencing neglect and financial abuse, and physical abuse, respectively. Conclusions: There is a high prevalence of elder abuse in Ugandan older adults, with neglect being the most common type of abuse. There is a need to design interventions for older adults at risk to prevent elder abuse from further escalating, where the present findings can be worthy of help.
**Order of Authors:**	Letizia Maria Atim, MD
	Mark Mohan Kaggwa, M.D
	Mohammed A Mamum
	Moses Kule
	Scholastic Ashaba

Powered by Editorial Manager® and ProduXion Manager® from Aries Systems Corporation

	Samuel Maling
**Additional Information:**	
**Question**	**Response**
**Financial Disclosure**<br><br>Enter a financial disclosure statement that describes the sources of funding for the work included in this submission. Review the submission guidelines for detailed requirements. View published research articles from *PLOS ONE* for specific examples.<br><br>This statement is required for submission and **will appear in the published article** if the submission is accepted. Please make sure it is accurate.<br><br>**Unfunded studies**<br>Enter: *The author(s) received no specific funding for this work.*<br><br>**Funded studies**<br>Enter a statement with the following details:<br>• Initials of the authors who received each award<br>• Grant numbers awarded to each author<br>• The full name of each funder<br>• URL of each funder website<br>• Did the sponsors or funders play any role in the study design, data collection and analysis, decision to publish, or preparation of the manuscript?<br>• **NO** - Include this sentence at the end of your statement: *The funders had no role in study design, data collection and analysis, decision to publish, or preparation of the manuscript.*<br>• **YES** - Specify the role(s) played.<br><br>* typeset	The author(s) received no specific funding for this work
**Competing Interests**<br><br>Use the instructions below to enter a competing interest statement for this submission. On behalf of all authors, disclose any competing interests that could be perceived to bias this work—acknowledging all financial support and any other relevant financial or non-financial competing interests.	The authors have declared that no competing interests exist.

This statement is required for submission and **will appear in the published article** if the submission is accepted. Please make sure it is accurate and that any funding sources listed in your Funding Information later in the submission form are also declared in your Financial Disclosure statement.<br><br>View published research articles from *PLOS ONE* for specific examples.<br><br>**NO authors have competing interests**<br><br>Enter: *The authors have declared that no competing interests exist.*<br><br>**Authors with competing interests**<br><br>Enter competing interest details beginning with this statement:<br><br>*I have read the journal's policy and the authors of this manuscript have the following competing interests: [insert competing interests here]*<br><br>* typeset	
**Ethics Statement**<br><br>Enter an ethics statement for this submission. This statement is required if the study involved:<br><br>• Human participants<br>• Human specimens or tissue<br>• Vertebrate animals or cephalopods<br>• Vertebrate embryos or tissues<br>• Field research<br><br>Write "N/A" if the submission does not require an ethics statement.<br><br>General guidance is provided below. Consult the submission guidelines for detailed instructions. **Make sure that all**	The study received ethics approval from the research ethics committee of Mbarara University of Science and Technology (approval number: MUSTREC#05/11-20). The director of MRRH granted permission to collect data from participants. All participants provided voluntary written informed consent at study enrollment.

Powered by Editorial Manager® and ProduXion Manager® from Aries Systems Corporation

**information entered here is included in the Methods section of the manuscript.**<br><br>**Format for specific study types**<br><br>**Human Subject Research (involving human participants and/or tissue)**<br>• Give the name of the institutional review board or ethics committee that approved the study<br>• Include the approval number and/or a statement indicating approval of this research<br>• Indicate the form of consent obtained (written/oral) or the reason that consent was not obtained (e.g. the data were analyzed anonymously)<br><br>**Animal Research (involving vertebrate animals, embryos or tissues)**<br>• Provide the name of the Institutional Animal Care and Use Committee (IACUC) or other relevant ethics board that reviewed the study protocol, and indicate whether they approved this research or granted a formal waiver of ethical approval<br>• Include an approval number if one was obtained<br>• If the study involved *non-human primates*, add *additional details* about animal welfare and steps taken to ameliorate suffering<br>• If anesthesia, euthanasia, or any kind of animal sacrifice is part of the study, include briefly which substances and/or methods were applied<br><br>**Field Research**<br><br>Include the following details if this study involves the collection of plant, animal, or other materials from a natural setting:<br>• Field permit number<br>• Name of the institution or relevant body that granted permission	
**Data Availability**<br><br>Authors are required to make all data underlying the findings described fully available, without restriction, and from the time of publication. PLOS allows rare exceptions to address legal and ethical	Yes - all data are fully available without restriction

concerns. See the PLOS Data Policy and FAQ for detailed information.<br><br>A Data Availability Statement describing where the data can be found is required at submission. Your answers to this question constitute the Data Availability Statement and **will be published in the article**, if accepted.<br><br>**Important:** Stating 'data available on request from the author' is not sufficient. If your data are only available upon request, select 'No' for the first question and explain your exceptional situation in the text box.<br><br>Do the authors confirm that all data underlying the findings described in their manuscript are fully available without restriction?	
**Describe where the data may be found in full sentences. If you are copying our sample text, replace any instances of XXX with the appropriate details.**<br><br>• If the data are **held or will be held in a public repository**, include URLs, accession numbers or DOIs. If this information will only be available after acceptance, indicate this by ticking the box below. For example: *All XXX files are available from the XXX database (accession number(s) XXX, XXX.*).<br>• If the data are all contained **within the manuscript and/or Supporting Information files**, enter the following: *All relevant data are within the manuscript and its Supporting Information files.*<br>• If neither of these applies but you are able to provide **details of access elsewhere**, with or without limitations, please do so. For example:<br><br>*Data cannot be shared publicly because of [XXX]. Data are available from the XXX Institutional Data Access / Ethics Committee (contact via XXX) for researchers who meet the criteria for access to confidential data.*	Data is provided as a supporting file

The data underlying the results presented in the study are available from (include the name of the third party and contact information or URL).<br>• This text is appropriate if the data are owned by a third party and authors do not have permission to share the data.<br><br>* typeset	
Additional data availability information:	

Manuscript Click here to access/download;Manuscript;Full manuscript.docx

Factors associated with elder abuse in rural Uganda: A hospital-based cross-sectional study

Letizia Maria Atim[1,2], Mark Mohan Kaggwa[1, 2,3*], Mohammed A. Mamum[4,5], Moses Kule[6], Scholastic Ashaba[1], and Samuel Maling[1]

Affiliations

1. Department of Psychiatry, Faculty of Medicine, Mbarara University of Science and Technology, Mbarara, Uganda.
2. African Centre for Suicide Prevention and Research, Mbarara, Uganda.
3. Department of Psychiatry and Behavioural Neurosciences, McMaster University, Canada
4. CHINTA Research Bangladesh, Savar, Dhaka 1342, Bangladesh.
5. Department of Public Health and Informatics, Jahangirnagar University, Savar, Dhaka 1342, Bangladesh.
6. Department of Psychiatry, Mbarara Regional Referral Hospital, Mbarara, Uganda.

***Corresponding authors**

Dr Mark Mohan Kaggwa, Email: kmarkmohan@gmail.com

Abstract

Background: There is a trend of a growing global population of older persons, especially in low-middle-income countries, and many older adults are vulnerable to abuse due to many factors, such as functional decline, increased elderly poverty, and neurocognitive disease, among others. Available literature shows that one in ten older persons is experiencing abuse. Abuse can impact the well-being of the elderly, decreasing their quality of life and leading to mental health challenges, morbidity, and mortality. Evidence on elderly abuse is vital to facilitate initiatives, but there are fewer studies on older adult abuse in Africa, particularly in Uganda. **Objective**: This study aimed to determine the prevalence of elderly abuse, the various types of elderly abuse and their associated factors in a Ugandan elderly sample. **Methods**: This was a hospital-based cross-sectional study among patients aged 60 years and above. Information on sociodemographic characteristics, functional impairment using the Barthel Index Scale, and elderly abuse severity using the Hwalek-Sengstock Elder Abuse Screening Test were collected. In addition, types of abuse, including neglect, physical, sexual, emotional, and financial abuse, were assessed using questions adapted from the US National Research Council on elder mistreatment monograph. Linear and logistic regression analyses were used to determine the factors associated with elderly abuse severity and the different types of abuse, respectively. **Results:** Overall, the prevalence of elder abuse was 89.0%. Neglect was the most common type of elderly abuse (86%), followed by emotional (49%), financial (46.8%), physical mistreatment (25%), and sexual (6.8%). About 30.4% of the abused elderlies experienced at least two forms of abuse. The factors associated with an increase in elderly abuse severity were having a secondary level of education and physical impairment. Moderate to severe functional dependence was associated with almost all forms of abuse. Individuals who reported the presence of a perpetrator were likely to experience neglect, emotional, and physical mistreatment. However, those who reported their perpetrators to the police had a higher likelihood of experiencing emotional abuse but were less likely to experience financial abuse. Emotional abuse was also associated with age above 80 years and attaining education (primary and secondary). Physical impairment and chronic medical conditions reduced the likelihood of experiencing neglect and financial abuse, and physical abuse, respectively. **Conclusions:** There is a high prevalence of elder abuse in Ugandan older adults, with neglect being the most common type of abuse. There is a need to design interventions for older adults at risk to prevent elder abuse from further escalating, where the present findings can be worthy of help.

Keywords: Elderly abuse severity, types of elderly abuse, Hwalek- Sengstock elder abuse screening test, functional dependency, geriatrics, neglect, mistreatment, sexual abuse, emotional abuse, physical abuse, financial abuse, and Uganda

1.0. Introduction

Due to several factors, such as higher life expectancy, lower fertility rates, improved healthcare systems, and longevity, the aged population is increasing globally [1]. About 1 billion people aged 60 years or above will increase to 1.4 billion and 2.1 billion by 2030 and 2050, respectively [2]. In low-and middle-income countries (LMICs), the expected population growth rates remain higher than in high-income countries [3], and 80% of the global older adults will be in LMICs by the year 2050 [2]. However, the increasing older adult population faces physical and mental health challenges, including elder abuse [4]. For instance, in Uganda, the elderly face multiple challenges, including poverty, poor health, unemployment, chronic ill-health, HIV/AIDS, lack of social security systems, low land productivity, political instability, low agricultural returns, and functional inability [5-8]. These challenges impact their quality of life and make them susceptible to neglect and abuse by their peers and family members [5, 9].

According to the World Health Organization (WHO), around 1 in 6 older adults have reported experiencing abuse each year [10]. Elder abuse can be in different forms, such as physical, sexual, emotional or psychological, financial, abandonment, neglect, and institutional abuse [5, 9]. Evidence from a systematic review of past-year abuse showed abuse among 15.7% of older persons [11]; emotional abuse was dominant at 11.6%, followed by financial (6.8%), then neglect at 4.2%, and sexual abuse least experienced at 0.9% [11]. In Europe, prevalence varies from 2.2% (Ireland) and 61.1% (Croatia), whereas, in Asia, the highest prevalence was reported in China (36.2%), and the lowest was in India at 14.0%, as per a systematic review [12]. In South Africa, men's and women's prevalences were 64.3% and 60.3%, respectively. Physical abuse was more common among men, while emotional, financial, and sexual abuse was more frequent among women [13]. Abused older adults present to the hospital with different signs and symptoms, including inadequately or usually unexplained locations of abrasions or bruises, lacerations, and burns [14]. Other signs are fractures in non-alcoholics, malnutrition and dehydration, pressure ulcers, sexually transmitted infections, and vaginal and rectal bleeding without a reasonable explanation [14].

Evidence has highlighted some of the factors associated with elder abuse across the globe, including (i) perpetrator factors such as mental illness, abuser dependency, and alcohol dependence; (ii) host factors such as dementia, physical dependency, living alone, or with spouse; and (iii) environmental factors such as social isolation and negative societal perceptions towards ageing [15]. In addition, psychiatric illness among older adults is an important cause of vulnerability to abuse, especially when it is comorbid with other risk factors such as physical frailty, sensory impairment, social isolation, and physical dependency [9]. Other prominent factors associated with elder abuse are caregiver burnout, stress problems, coping with perpetrator childhood abuse, and relationship problems such as intimate partner violence [16].

Elder abuse is also associated with various adverse health outcomes, including physical and psychological effects that have long-term effects such as depression, anxiety, and post-traumatic disorder [17]. It also can pose significant complications, such as premature death. For example, community-dwelling middle-aged and older women who reported prior physical, verbal, or both types of abuse had significantly higher adjusted mortality risk than

women who did not report abuse [18]. In addition, reported and corroborated elder mistreatment is associated with shorter survival in both women and men [19]. Abused elders also face complications such as post-traumatic stress disorder, poorly controlled chronic diseases such as hypertension, diabetes, heart disease, decreased quality of life, and loss of trust or quality of relationships [20]. Furthermore, elder abuse poses significant complications to society, like the cost of care for victims, the use of community legal and law enforcement resources, and the burden on nursing facilities [20].

Despite the dangers of elder abuse, information on the prevalence, types, and factors associated with elder abuse in Africa is sparse. Therefore, the present study aimed to determine the prevalence of elderly abuse, the various types of abuse, and their associated factors in a Ugandan elderly sample attending tertiary hospital outpatient clinics.

2.0. Methods

2.1. Study design, setting and population

This study was a cross-sectional descriptive study done in February 2021 among older adults aged 60 years and above attending a referral hospital outpatient clinic in Uganda that attends to approximately 600 older adults every month [6, 21]. After receiving care for various ailments, elders were recruited from the outpatient departments by convenience sampling. Based on the Mini-Mental Status Examination, we excluded elders with severe neurocognitive impairment (a score of 17 and below). We thus recruited 363 participants.

2.2. Data collection

The research assistants identified participants who met the inclusion criteria and fast-tracked them to receive medical care after completing the interview. Then, they collected data and interviewed each participant for a minimum of 40 minutes using translated questionnaires.

2.3 Study variables and measures

2.3.1 Basic Information

This study collected basic sociodemographic information such as gender, age, address (rural vs urban), occupation (previous employment formal vs informal; currently inactive or active at the employment), marital status, level of education (none, primary, secondary, and post-secondary education), and type of housing they are currently staying in (public/government-owned, rental, personally owned) – these show the amount of autonomy one has on their property. In addition, we asked about chronic medical illnesses such as cancer, HIV, diabetes, hypertension, etc., and physical impairment such as blindness, lame, and crippled.

2.3.2 Elderly Abuse

The Hwalek-Sengstock Elder Abuse Screening Test (HS-EAST) was used to screen for elderly abuse severity [22]. The scale consists of 15 items and three conceptual categories. These three conceptual categories include “overt violation of personal rights and direct abuse” (items 4, 9, 10, 11, 15) and “characteristics of elder that make him or her vulnerable to abuse” (items

1, 3 and 6) and characteristics of potentially abusive situations (items 2, 5, 7, 8, 12, 13 and 14) [23]**.** It is scored by summing responses from each item (Yes/No), where 'Yes' - scored one and 'No' - scored 0. Possible scores range from 0 to 15, where a higher score represents higher exposure to elderly abuse. Based on previous studies, the HS-EAST had a cutoff of 3 and above with a sensitivity and specificity of 82.8% and 84.5%, respectively, for elderly abuse; and a reported Cronbach alpha of 0.741 [24]. In this study, the Cronbach alpha was 0.78.

In addition to elderly abuse, different types of abuse experienced in the past six months (such as physical, emotional, and financial abuse) were assessed using questions adapted from the National Research Council on elder mistreatment monograph, **Supplementary File 1** [15]. Lastly, the older adults who preferred to report also gave information about the perpetrators.

2.3.3. Functional Impairment

The level of functionality was tested using the Barthel Index (BI). The BI is composed of 10 items with varying weights [25]. The tool has two items assessing personal care (wash face, comb hair, shave, and clean teeth) and bathing evaluated with a 2-score scale (0 and 5 points); 6 items regarding feeding, getting onto and off the toilet, ascending and descending stairs, dressing, controlling bowels, and controlling bladder are evaluated with a 3-score t scale (0, 5, and 10 points); and two items regarding moving from wheelchair to bed and returning, and walking on a level surface are evaluated with a 4-score scale (0, 5, 10, and 15 points). The BI is a cumulative score calculated by summing each item's score. The BI scores are multiples of 5 with a range of 0 (completely dependent) to 100 (independent in basic activities of daily living (ADL)). Higher scores represent a higher degree of independence [25]. The score is categorized into 5 groups 0 – 20 = total dependency, 21 - 60 = severe dependency, 61 – 90 = moderate dependency, 91 – 99 = slight dependency, and 100 = complete independence. The Cronbach alpha for BI is 0.81 [25]. For this study, the Cronbach alpha was 0.88.

2.5. Ethical considerations

Formal ethical approval for this study was obtained from the Mbarara University of Science and technology research ethics committee (#05/11-20). Administrative clearance to conduct the study was obtained from the tertiary hospital director. Participants who agreed to participate in this study appended their signature or thumbprint (for those who could not read or write) on the consent form. The consent form translated to the local language (Runyankole) was read aloud to individuals who could not read and write, signed in the presence of a trusted witness (fluent in reading and writing) who countersigned. Participants diagnosed with severe neurocognitive impairment were referred to the psychiatry department for investigation and further management. All participants were interviewed in a private room away from their caretakers and other patients to maintain privacy. Counselling was provided to all participants who experienced any form of abuse by a trained study psychologist with experience in dealing with domestic violence. Individuals who experienced abuse were referred to the district probation office – an office responsible for managing cases of violence and abuse at the district level, for further management of the situation.

2.6. Data analysis

Data was initially entered into an excel sheet and then exported to STATA 16.0 for the final analysis. Chi-square tests were performed to determine significant differences between individuals who experienced various forms of abuse and those who did not. For elder abuse severity based on the HS-EAST, we ran a *t*-test and ANOVA for the elderly abuse severity. Linear regression analysis determined the factors associated with elder abuse severity. A back stepwise multivariable linear regression was built after testing for collinearity to adjust for a cofounder. In addition, logistic regression was used to determine the factors associated with the different types of elderly abuse (neglect, physical, sexual, emotional, and financial abuse). The significant level was set at less than 5% for a 95% confidence interval.

3. Results

3.1. Characteristics of the Participants

A total of 363 participants were included in this study, where 57% (n=208) of them were females. The mean age of the participants was 67.08 (SD=6.66) years, and most belonged to the age group 60–69 years (69.0%). Most participants came from a rural setting (71%, n=259),72.3% had a comorbid medical condition, and 44.4% had a physical impairment (**Table 1**).

3.2. Elderly Abuse

Mean elderly abuse severity was 6.0 (SD=2.67) out of 15 at the HS-EAST, whereas 89.0% of elderly had met the cutoff point 3 and above the HS-EAST. In addition, these participants experienced more characteristics of potentially abusive situations, mean of 2.99 (1.47; out of 7), followed by experiencing an overt violation of personal rights and direct abuse, 2.05 (1.41; out of 5).

Participants with physical impairment had higher elderly abuse severity mean scores than those without physical impairments (6.56% vs 5.90%, t=-3.71, p<0.001). Also, elders' who stayed in government-owned housing had higher elderly abuse severity mean scores (F=4.15, p=0.016), and those whose highest level of education was post-secondary had the lowest elderly abuse severity score as per the education level (F=5.03, p=0.002) (**Table 2**).

3.2.1. Factors Associated with Elderly Abuse Severity

At bivariate analysis, the factors that increased elderly abuse severity were: being divorced or separated, previously informally employed and currently still active, having a secondary level of education, staying in a government-owned house, and having a physical impairment. These factors were tested for collinearity and had VIFs below 3; the mean VIF was 1.14. They were included in the final model using backwards stepwise modelling. There was evidence of homoskedasticity with a Cook-Weisberg test p-value of 0.119. The final model could explain 9.7% of elderly abuse severity. At multiple variate analyses, having a secondary level of education statistically significantly increased elderly abuse severity by 0.97, and having a physical impairment increased the elderly abuse severity by 0.83 (**Table 2**).

3.3. Prevalence of Different Types of Abuse

The prevalences of the different types of abuse were 86.3%, 49.0%, 46.8%, 22.2%, and 6.51, for neglect, emotional, financial, physical, and sexual abuse, respectively. About 30.4% of the participants experienced any two types of abuse (**Figure 1**), and a majority (66.6%, n = 243) experienced both physical mistreatment and neglect (**Table 3**). Experiencing physical mistreatment and emotional abuse had a low significant correlation (r^2=0.35). The elderly abuse severity significantly correlated negligibly with emotional abuse and physical mistreatment. However, the other combinations with significant correlations also had negligible correlations. For details, see **Table 4**.

3.3.1. Factors Associated with Emotional Abuse

Previously informally employed, currently retired, and unemployed participants were more emotionally abused (χ^2=9.08, p=0.028). Emotional abuse was less among individuals with a chronic medical condition than those with no chronic medical condition, and the difference was statistically significant (45.8% vs 57.4%, χ^2=3.93, p=0.047). Moderately functionally dependent elders were more emotionally abused (65.2%) than those who were more independent (43.8% and 46.0% for a slight/little dependent and completely independent, respectively) or severely functionally dependent (36.4%) (χ^2=9.35, p=0.025) (**Supplementary file 2)**.

At bivariate analysis (**Supplementary file 3**), the factors associated with the increase in the likelihood of experiencing emotional mistreatment were being moderately functional dependent, having to report the perpetrator to the police, or acknowledging the presence of a perpetrator. However, elders with a chronic illnesses were less likely to experience emotional abuse. The factors were tested for collinearity; all had individual VIFs of less than three, and the mean VIF was 1.04. AT multivariate logistic regression, the factors associated with experiencing emotional mistreatment were having reported having a perpetrator (aOR=7.94, 95% CI: 3.71-17.06, p<0.001), having reported the perpetrator to the police (aOR=3.33, 95% CI: 1.25-8.86, p=0.016), and being moderately functionally dependent (aOR=1.85, 95% CI: 1.01-3.78, p=0.047) (**Table 5**). This final model had a specificity of 88.71%, a sensitivity of 41.90%, a positive predictive value (PPV) of 78.13%, a negative predictive value (NPV) of 61.34%, and was correctly classified as 65.75% of individuals with who experienced emotional elderly abuse. The goodness of fit p-value was 0.838 for the included four variables.

3.3.2. Factors Associated with Physical Abuse

The largest statistical difference was between individuals who reported perpetrators and those who did not, with those who reported experiencing more physical abuse than their counterparts (50.0% vs 16.0%; χ^2=26.8, p<0.001). Older adults aged 80 years and above experienced more physical abuse than those younger (43.5% vs 22.6% and 15.6% for 60 -69 years and 70 – 79 years, respectively) (χ^2=8.36, p=0.015). All individuals who never married experienced physical abuse, whereas 21.7%, 22.4%, and 20.7% of individuals who were either cohabiting/married, divorced/separated, or widowed, respectively, experienced physical abuse (χ^2=10.67, p=0.014). Older adults having a chronic medical illness were statistically less physically abused (17.4%) compared to those with no chronic medical condition (34.6%) (χ^2=12.6, p-value <0.001). Older adults with moderate functional dependence were the main

culprit for physical abuse (37.7%) compared with individuals who were more functionally independent (13.6% and 19.8% for slightly dependent and completely independent, respectively) (χ^2=13.05, *p*=0.005) (**Supplementary file 2**).

At bivariate analysis, individuals with chronic medical illnesses were less likely to experience physical abuse (**Supplementary file 3**). However, those above 80 years had reported the perpetrator to the police or acknowledged their presence, and being moderate functionally dependent increased the likelihood of experiencing physical mistreatment at bivariate analysis. These factors were tested for collinearity, and they all had VIFs below 3, with a mean VIF of 1.02, and consequently, they were included in the final model. The model had a sensitivity of 28.40%, specificity of 95.07%, a PPV of 62.16%, and NPV of 82.32%, and correctly classified 80.27% of experiencing physical mistreatment. The goodness-of-fit p-value was 0.639 and included five variables. In the multivariable logistic regression, the factors associated with experiencing abuse were having reported the presence of a perpetrator (aOR=5.12, 95% CI: 2.79-9.44, *p*<0.001) and an elderly who has moderate functional dependence (aOR=2.44, 95% CI: 1.13-4.08, *p*=0.019). However, chronic physical medical conditions reduced the likelihood of experiencing physical mistreatment (aOR=0.40, 95% CI: 0.23-0.71, *p*=0.002) (**Table 5**).

3.3.3. Factors Associated with Neglect

There was a statistical difference between neglect and functional dependence (χ^2=18.78, *p*<0.001); neglect was more among elders with higher levels of dependence. Participants with physical impairment were statistically less neglected than those without physical impairment (79.0% vs 92.1%, χ^2=13.09, *p*<0.001). Elders who reported having a perpetrator (95.4%) were statistically neglected more compared to those who declined (84.3%) (χ^2=5.71, *p*=0.017) (**Supplementary file 2**).

At bivariate analysis (**Supplementary file 3**), neglect was associated with having the reported presence of a perpetrator and an increasing level of functional dependence; that is, those with moderate functional dependence have a higher likelihood of experiencing neglect than those with slight functional dependence. Staying in publically owned/government housing and having a physical impairment significantly reduced the likelihood of experiencing neglect. These factors had a mean VIF of 1.01, and all their VIFs were below three at testing for collinearity. At multivariate analysis (**Table 5**), having physical impairment reduced the likelihood of neglect (aOR=0.27, 95% CI: 0.14-0.52, *p*<0.001). However, individuals who reported the presence of a perpetrator and those with slight or moderate functional dependence were likely to experience neglect. The likelihood of experiencing neglect was highest in individuals with moderate functional dependence (aOR=9.33, 95% CI: 2.15–40.42, *p*=0.003). This final model had a sensitivity of 100%, specificity of 0%, PPV of 85.88%, and correctly classified 85.88% of neglect experienced by the older adults.

3.3.4. Factors Associated with Financial Abuse

There was a statistical difference between financial abuse and the following variables: age, gender, dwelling area, employment status, level of education, presence of chronic illness, presence of physical impairment, history of a police report, and functional dependence.

Participants aged 80 and above were statistically more financially abused (91.3%) compared with younger participants (43.2% and 45.6% for 60-69 and 70–79, respectively) (χ^2=19.6 p<0.001). Male participants suffered more financial abuse than females (54.8% vs 40.9%; χ^2=6.95, p=0.008). Urban dwellers statistically suffered more elderly abuse than rural dwellers (55.7% vs 43.2%; χ^2=4.66, p=0.031). For details, see **Supplementary file 2**.

At bivariate analysis (**Supplementary 3**), having experienced financial abuse was associated with staying in an urban setting, male gender, age above 80 years, level of education (primary and tertiary), and severe functional dependence. The likelihood was reduced among individuals with chronic physical medical conditions, those with physical impairment, and those who reported their perpetrators to the police. The factors significant at bivariate analysis were tested for collinearity; all had a VIF less than 3 with a mean VIF of 1.08. All were included in the final multivariate logistic model whose sensitivity was 57.31%, specificity of 76.29%, PPV of 68.06%, NPV of 66.97%, and correctly classified 67.40% of financial abuse. Physical impairment and reporting the perpetrator to the police were significant factors that reduced the likelihood of financial abuse. However, age above 80 (aOR=4.24, 95% CI: 2.37-53.21, p=0.002), having primary (aOR=2.01 95% CI: 1.08-3.72, p=0.0027) or secondary (aOR=3.65, 95% CI: 1.48-9.02, p=0.005) as highest level of education, and being severely functional dependent (aOR=8.95, 95% CI: 1.57-51.08, p=0.014) significantly increased the likelihood of experiencing financial abuse. (**Table 5**)

3.3.5. Factors Associated with Sexual Abuse

The was no gender difference between the level of sexual abuse experienced (**Supplementary file 2**). No factor was associated with sexual abuse. (**Supplementary file 3**)

4. Discussion

In this survey of older adults aged 60 years and above attending an outpatient department of a tertiary hospital in Southwestern Uganda, the prevalence of elderly abuse (cutoff of 3 out of 15 at the HS-EAST) was high (89.0%). The factors associated with an increase in the elderly abuse severity were having a secondary level of education and having a physical impairment. However, the prevalence of the various types of elder abuse was highest with neglect (86.0%) and lowest with sexual abuse (6.8%), and about 30.4% of the abused experienced at least two forms of abuse, especially physical mistreatment and neglect. Moderate to severe functional dependence was associated with all forms of abuse apart from sexual abuse. Individuals who reported a perpetrator were likely to experience neglect, emotional, and physical mistreatment. However, those who reported their perpetrators to the police had a higher likelihood of experiencing emotional abuse but were less likely to experience financial abuse. Emotional abuse was also associated with age above 80 years and attaining education (primary and secondary). Physical impairment and chronic medical conditions reduced the likelihood of experiencing neglect and financial abuse, and physical abuse, respectively.

4.1. Elderly Abuse

The prevalence of elderly abuse (89.0%) was much higher than that estimated from India (50.2%) [26], the USA (35.0%) [27], China (36.2%) [28, 29], and Iran (38.5%) [30] that used

a similar tool - the HS-EAST. The high prevalence in this study may be because we recruited a hospital sample that some of the victims visited to treat complications of elderly abuse such as injuries, illness, and mental health challenges [31]. In addition, the current study was in a country with limited laws and follow-up on elderly abuse, on top of a culture system diluted to the extent of the young or their children disrespecting the elderly [5].

In this study, having a secondary level of education (more than eight years of study in Uganda) increased the elderly abuse severity. A finding contrary to other study findings, which report that a history of education for more than eight years reduced elderly abuse severity [32]. Older adults with higher education levels can claim their rights, easily report abuse to responsible bodies, and even get more social respect [32]. However, recently older adults in Uganda have been disrespected by the young due to rapid cultural change [5], which puts many older adults at risk of abuse. In addition, the country is a low-income country with challenges such as unemployment, poverty, and a high fertility rate - many of the younger individuals financially exploit the weak and vulnerable elderly, especially the educated who might have been financially stable and have accumulated some assets over time so that they grab or sell their assets and earn some money [5]. No wonder these older adults with a higher level of education are at a higher likelihood of being financially abused in our study.

Also, older adults with physical impairment had more severe elderly abuse in this study, a finding similar to that reported in the previous studies [13, 28]. This finding is not surprising as people with disabilities depend on others, especially in fulfilling their daily activities [33]. In addition, older adults with physical impairment may not defend themselves or report their perpetrators to the police due to their inability to fight for themselves and fear of being neglected.

4.2. Financial Abuse

Almost half of the participants experienced financial abuse, which was higher than reported in other African countries, including Nigeria (13.1%) [34], Egypt (27%) [35], and South Africa (24.4%) [13]. Despite all these African countries belonging to the LMICs, Uganda has one of the lowest Gross Domestic Product [36]; thus, its citizens experience a higher poverty level, leading them to abuse financially stable or dependent older adults. Financially stable or dependent older adults become a primary target by some poor youth due to their vulnerabilities due to old age. Financial abuse in this study was associated with increasing age above 80 years because individuals above 80 years are more vulnerable to abuse. After all, they are at higher risk of neurocognitive disorders and functional dependence, which makes them unable to manage their finances or forget to request financial assistance [6]. They may also be more likely to make poor financial decisions and fall victim to fraudulent deals [37]. An increase in functional dependency with age is a major factor that increases the likelihood of almost all types of abuse except sexual abuse due to increased vulnerability and need for help in most of their daily activities. An elderly with a high level of functional dependence causes significant care burden stress, leading to an increased likelihood of abuse [38].

The likelihood of being financially abused was reduced when the older adult reported the known victim to the police. Reporting the perpetrators to the police, a method suggested by the CDC to reduce abuse, was effective in this study [4]. A perpetrator reported to the police

may be punished for their acts, for instance, taken away from the community or the victims' premises, thus, reducing further abuse. In addition, reporting a perpetrator to the police will make the community aware of the perpetrating persons, which will protect the potential victims. This community support may provide emotional and social support, thus reducing the likelihood of experiencing emotional abuse and other forms of elderly abuse. In Uganda, the community considers abuse of an older adult taboo [5]. Disrespecting or abusing an older adult with a disability is considered a worse offence by the community. Due to this respect and possible fear of the community reaction, many perpetrators do not abuse older adults with physical disabilities. The respect they give individuals with a disability may be responsible for them being less likely to experience financial abuse and neglect since every community member expects one to take good care of them.

4.3. Physical Abuse and Mistreatment

The prevalence of physical abuse (22.2%) among the older adults in this study was higher than that reported by most countries in a systematic review of 20 studies (0.2%–4.9%) [39]. However, studies included in the review by Pillemer et al. (2016) measured physical abuse using the conflict tactic scale that predominantly looks at intimate partner violence, excluding abuse from other possible perpetrators such as children, neighbours, and others; considered in our study. Thus, a large difference between the reported prevalence rates. On the other hand, the current study's prevalence of physical abuse was less than that reported by a Nigerian study (47.0%) [40]. The difference may be due to cultural belief differences between the two countries and associating the older adults with witchcraft due to their appearance (wrinkled skin, gnarled hands, and yellow eyes) [41]. In addition, the Nigerian study classified physical neglect as part of physical abuse, which could have led to a higher prevalence since neglect is a commonly reported type of elderly abuse [42].

Like other types of elderly abuse, experiencing physical abuse was associated with increased functional dependence. The individuals who reported the presence of perpetrators had a higher likelihood of physical abuse; this may be due to the demeaning nature of physical abuse and the desire to get help from others (i.e., reporting is an act of asking for help). However, individuals with chronic physical medical conditions were less likely to experience physical abuse since they looked too sick. Their perpetrators may not abuse them due to fear of them possibly killing them. It contradicted other studies where chronic diseases were associated with physical abuse [43].

4.4. Emotional Abuse

Nearly half of all older adults in this study experienced emotional abuse, comparable to findings in South Africa, Nigeria, and Egypt [13, 35, 44]. These findings could be because there are ways of addressing older adults in traditional African society, and a deviation from this norm is easily noticeable and considered disrespectful [34]. However, the prevalence of emotional abuse was much higher than that reported by Cadmus and Owoaje (2012) in Nigeria among women without psychiatric illnesses [34]. Despite psychiatric illnesses such as depression being associated with or a complication of emotional abuse [17]. The psychological suffering associated with emotional abuse was associated with reporting the

presence of a perpetrator to the research team and police in an attempt to seek emotional relief.

4.5. Neglect

As reported by other researchers in Africa [26, 35], neglect was the most dominant type of abuse. This high prevalence could be attributed to the loss of caregivers due to the HIV scourge in Africa or the migration of family members to urban areas compounded by the loss of extended family ties leaving the elders to fend for themselves and hence feel neglected [45]. Poverty is another factor that could explain high-rate neglect, as caregivers cannot take care of their own families, let alone older adults [46]. In addition, this study recruited older adults seeking care in hospitals with a high probability of having chronic illnesses, which put them at an increased likelihood of neglect due to limitations in activities of daily living and their associated high financial demands from the caregivers [16, 46, 47]. On the other hand, participants with physical impairment had a reduced likelihood of being neglected. Since a physical impairment limits an older adult's ability to manage daily life activities, putting them at risk of self-neglect [48, 49], this vulnerability makes caregivers present. The caregivers take care of them since not caring for the physically impaired is not culturally accepted [5].

4.6. Sexual abuse

Sexual abuse was the least prevalent type, similar to other studies [11, 13, 34, 42, 50, 51]. In the current study, there was no factor associated with sexual abuse. However, studies in South Africa and Canada have reported that women are at a higher risk of sexual abuse than males [13, 51]. In Africa, sexual abuse of older persons seldom occurs, and when it occurs, it may be ritual related to getting rich or getting special spiritual powers [52, 53]. In addition, sexual abuse of older adult women is not expected as ageist perceptions produce a taboo around considering sexual relations with older people [54]. Furthermore, older persons who are sexually abused may not report sexual abuse due to feelings of shame [55].

5. Limitations

First, the study was cross-sectional in design, so causality between elder abuse and its predictors can not be determined. Second, the study was hospital-based; therefore, elders at higher risk of abuse were sampled, leading to overestimating the prevalence rates. Lastly, the tool used to determine abuse was not adapted in Uganda; therefore, it could have been estimated as higher than the actual prevalence.

6. Conclusions

This study highlights a high prevalence of elder abuse in Uganda, with the most common forms of abuse being neglect, emotional abuse, and financial abuse. In addition, our study showed that women who were physically impaired, separated, and widowed had severe elderly abuse. There is, therefore, a need to design interventions to prevent vice in these vulnerable groups.

7. Declarations

Ethical approval and consent of participants: The study was conducted in accordance with the Declaration of Helsinki. The study received ethics approval from the research ethics committee of Mbarara University of Science and Technology (approval number: MUSTREC#05/11-20). The director of MRRH granted permission to collect data from participants. All participants provided voluntary written informed consent at study enrollment.

Consent for publication: Not applicable

Availability of data and materials: The datasets used in analysis during the current study are available as a supplementary file 4.

Competing interests: The authors declare that they have no competing interests.

Funding: The study was funded by the Kayanja Fellowship Program, Mbarara, Uganda

Authors' contribution: All authors partook in the conception and design of the study. MMK, MK, LMA, SA, & SM implemented the project and collected data, whereas SM and SA supervised the project. MMK and MAM performed formal analysis, and all authors made data interpretations. LMA drafted the initial version of the manuscript. All authors approved the final version of the manuscript.

Acknowledgement: We acknowledge the research assistants Franklin Kakuru, Brenda Nabatanza, Badru Kayongo, Innocent Arinaitwe, and Sarah Maria Najjuka. Mbarara regional referral hospital provided a conducive environment for data collection, and without the participants, this data would not have been obtained.

8. Reference

1. United States Census Bureau: **An Aging World: 2015**. In: *An Aging World: 2001.* Edited by Kebin K, Victoria AV, vol. 2021. US: US Department of Commerce; 2001. Retrieved on 29th December, 2021 from:: https://www.census.gov/library/publications/2001/demo/p95-01-1.html
2. World Health Organisation. (2021). Ageing. Retrieved on 29th December, 2021 from: https://www.who.int/health-topics/ageing
3. Afshar S, Roderick PJ, Kowal P, Dimitrov BD, Hill AG: **Multimorbidity and the inequalities of global ageing: a cross-sectional study of 28 countries using the World Health Surveys**. *BMC Public Health* 2015, **15**(1):776. https://doi.org/10.1186/s12889-015-2008-7.
4. Center for Disease Control and Prevention. (2021). **Preventing elder abuse**. Retrieved on 29th December, 2021 from: https://www.cdc.gov/violenceprevention/elderabuse/fastfact.html
5. Wamara CK, Bennich M, Strandberg T: **Missing voices: older people's perspectives on being abused in Uganda**. *Journal of Elder Abuse & Neglect* 2021, **33**(4):288-310. https://doi.org/10.1080/08946566.2021.1970682.
6. Atim LM, Kaggwa MM, Mamun MA, Ashaba S, Maling S: **Prevalence of severe neurocognitive impairment and its association with socio-demographics and**

functionality among Ugandan older persons: a hospital-based study. *Clinical interventions in aging* 2021, **16**:1415-1425. https://doi.org/10.2147/cia.S319891.

7. Kabuye R, Mukasa N: **Older people's conceptualisation of poverty and their experiences of government programmes in Uganda**. *Development in Practice* 2019, **29**(3):349-359. https://doi.org/10.1080/09614524.2018.1552659.
8. Najjumba-Mulindwa, I. (2003, 2003). **Chronic poverty among the elderly in Uganda: perceptions, experiences and policy issues.** Retrieved on 29th December, 2021 from: https://assets.publishing.service.gov.uk/media/57a08cdde5274a31e00014dc/Najjumba-Mulindwa.pdf
9. Cooper C, Livingston G: **Mental health/psychiatric issues in elder abuse and neglect**. *Clinics in geriatric medicine* 2014, **30**(4):839-850. https://doi.org/10.1016/j.cger.2014.08.011.
10. World Health Organisation. (2017). **Elder abuse.** Retrieved on 29th December, 2021 from: https://www.who.int/news-room/fact-sheets/detail/elder-abuse
11. Yon Y, Mikton CR, Gassoumis ZD, Wilber KH: **Elder abuse prevalence in community settings: a systematic review and meta-analysis**. *The Lancet Global Health* 2017, **5**(2):e147-e156. https://doi.org/10.1016/S2214-109X(17)30006-2.
12. Dong XQ: **Elder abuse: systematic review and implications for practice**. *Journal of the American Geriatrics Society* 2015, **63**(6):1214-1238. https://doi.org/10.1111/jgs.13454.
13. Bigala P, Ayiga N: **Prevalence and predictors of elder abuse in Mafikeng Local Municipality in South Africa**. *Asian Population Studies* 2014, **28**:463-474. https://doi.org/10.11564/28-1-500.
14. Wang XM, Brisbin S, Loo T, Straus S: **Elder abuse: an approach to identification, assessment and intervention**. *CMAJ : Canadian Medical Association journal = journal de l'Association medicale canadienne* 2015, **187**(8):575-581. https://doi.org/10.1503/cmaj.141329.
15. National Research Council Panel to Review, Risk and Prevalence of Elder Abuse and Neglect: **The national academies collection: reports funded by National Institutes of Health**. In: *Elder Mistreatment: Abuse, Neglect, and Exploitation in an Aging America.* edn. Edited by Bonnie RJ, Wallace RB. Washington (DC): National Academies Press (US) Copyright © 2003, National Academy of Sciences.; 2003.
16. Storey JE: **Risk factors for elder abuse and neglect: a review of the literature**. *Aggression and Violent Behavior* 2020, **50**:101339. https://doi.org/10.1016/j.avb.2019.101339.
17. Dong X, Chen R, Chang ES, Simon M: **Elder abuse and psychological well-being: a systematic review and implications for research and policy - a mini review**. *Gerontology* 2013, **59**(2):132-142. https://doi.org/10.1159/000341652.
18. Baker MW, Sugar NF, Eckert LO: **Sexual assault of older women: Risk and vulnerability by living arrangement**. *Sexuality Research and Social Policy Journal of NSRC* 2009, **6**(4):79-87. https://doi.org/10.1525/srsp.2009.6.4.79.
19. Lachs MS, Pillemer K: **Abuse and neglect of elderly persons**. *The New England journal of medicine* 1995, **332**(7):437-443. https://doi.org/10.1056/nejm199502163320706.
20. Johnson MJ, Fertel H. **Elder abuse**. [Updated 2021 Aug 12]. In: StatPearls [Internet]. Treasure Island (FL): StatPearls Publishing; 2021 Jan-. Available from:

https://www.ncbi.nlm.nih.gov/books/NBK560883/Jones, H., & Powell, J. L. (2006). Old age, vulnerability and sexual violence: implications for knowledge and practice. *International Nursing Review, 53*(3), 211-216. https://doi.org/10.1111/j.1466-7657.2006.00457.x..

21. Kaggwa MM, Acai A, Rukundo GZ, Harms S, Ashaba S: **Patients' perspectives on the experience of absconding from a psychiatric hospital: a qualitative study**. *BMC Psychiatry* 2021, **21**(1):371. https://doi.org/10.1186/s12888-021-03382-0.
22. Neale AV, Hwalek MA, Scott RO, Sengstock MC, Stahl C: **Validation of the Hwalek-Sengstock Elder Abuse Screening Test**. *Journal of Applied Gerontology* 1991, **10**(4):406-418. https://doi.org/10.1177/073346489101000403.
23. Ozmete E: **The Hwalek-Sengstock Elder Abuse Screening Test: the adaptation study into Turkish/Hwalek-Sengstock Yasli Istismari Tarama Testi: Turkceye uyarlama calismasi**. *Anadolu Psikiyatri Dergisi* 2016, **17**(S1):45-53.
24. Aminalroaya R, Alizadeh-Khoei M, Hormozi S, Sharifi F, Taati F: **Screening for elder abuse in geriatric outpatients: reliability and validity of the Iranian version Hwalek-Sengstock Elder Abuse Screening Test (H-S/EAST)**. *J Elder Abuse & Neglect* 2020, **32**(1):84-96. https://doi.org/10.1080/08946566.2020.1719564.
25. Mahoney FI, Barthel DW: **Functional evaluation: the Barthel Index**. *Maryland state medical journal* 1965, **14**:61-65.
26. Saikia, A. M., Mahanta, N., Mahanta, A., Deka, A. J., & Kakati, A. (2015). **Prevalence and risk factors of abuse among community dwelling elderly of Guwahati City, Assam.** *Indian journal of community medicine : official publication of Indian Association of Preventive & Social Medicine, 40*(4), 279-281. https://doi.org/10.4103/0970-0218.164406..
27. Fulmer T, Strauss S, Russell SL, Singh G, Blankenship J, Vemula R, Caceres B, Valenti M, Sutin D: **Screening for elder mistreatment in dental and medical clinics**. *Gerodontology* 2012, **29**(2):96-105. https://doi.org/10.1111/j.1741-2358.2010.00405.x.
28. Wu L, Chen H, Hu Y, Xiang H, Yu X, Zhang T, Cao Z, Wang Y: **Prevalence and associated factors of elder mistreatment in a rural community in People's Republic of China: a cross-sectional study**. *PloS one* 2012, **7**(3):e33857-e33857. https://doi.org/10.1371/journal.pone.0033857.
29. Ramalingam A, Sarkar S, Premarajan KC, Rajkumar RP, Subrahmanyam DK: **Prevalence and correlates of elder abuse: a cross-sectional, community-based study from rural Puducherry**. *The National medical journal of India* 2019, **32**(2):72-76. https://doi.org/10.4103/0970-258x.275344.
30. Hosseinkhani Z, Moradi Z, Khodamoradi F: **Elder abuse: screening in Iranian families**. *Medical journal of the Islamic Republic of Iran* 2017, **31**:126. https://doi.org/10.14196/mjiri.31.126.
31. Rosen T, Mehta-Naik N, Elman A, Mulcare MR, Stern ME, Clark S, Sharma R, LoFaso VM, Breckman R, Lachs M *et al*: **Improving quality of care in hospitals for victims of elder mistreatment: development of the vulnerable elder protection team**. *Joint Commission journal on quality and patient safety* 2018, **44**(3):164-171. https://doi.org/10.1016/j.jcjq.2017.08.010.

32. Skirbekk V, James KS: **Abuse against elderly in India – the role of education**. *BMC Public Health* 2014, **14**(1):336. https://doi.org/10.1186/1471-2458-14-336.

33. Sathya T, Premkumar R: **Association of functional limitations and disability with elder abuse in India: a cross-sectional study**. *BMC Geriatrics* 2020, **20**(1):220. https://doi.org/10.1186/s12877-020-01619-3.

34. Cadmus EO, Owoaje ET: **Prevalence and correlates of elder abuse among older women in rural and urban communities in South Western Nigeria**. *Health care for women international* 2012, **33**(10):973-984. https://doi.org/10.1080/07399332.2012.655394.

35. El-Khawaga G, Eladawi N, Abdel-Wahab F: **Abuse of rural elders in Mansoura districts, Dakahlia, Egypt: prevalence, types, risk factors, and lifestyle**. *Journal of interpersonal violence* 2021, **36**(5-6):Np2868-np2882. https://doi.org/10.1177/0886260518767900.

36. Simona , V., & Statista. (2021). **GDP per capita of African countries 2021**. Retrieved on 29th December, 2021, from: https://www.statista.com/statistics/1120999/gdp-of-african-countries-by-country/

37. Wood S, Lichtenberg PA: **Financial capacity and financial exploitation of older adults: Research findings, policy recommendations and clinical implications**. *Clinical Gerontology* 2017, **40**(1):3-13. https://doi.org/10.1080/07317115.2016.1203382.

38. Kohn R, Verhoek-Oftedahl W: **Caregiving and elder abuse**. *Medicine and health, Rhode Island* 2011, **94**(2):47-49.

39. Pillemer K, Burnes D, Riffin C, Lachs MS: **Elder abuse: global situation, risk factors, and prevention strategies**. *The Gerontologist* 2016, **56 Suppl 2**(Suppl 2):S194-205. https://doi.org/10.1093/geront/gnw004

40. Akpan ID, Umobong ME: **An assessment of the prevalence of elder abuse and neglect in Akwa Ibom state, Nigeria**. *Developing Country Studies* 2013, **3**:8-14. Retrieved on 29th December, 2021, from: https://www.iiste.org/Journals/index.php/DCS/article/view/5493.

41. Phakathi NN: **Exploring elder abuse among clinic attendees in a selected Durban hospital**. *Masters dissertation.* South Africa: University of KwaZulu-Natal; 2011. Retrieved on 29th December, 2021, from: https://ukzn-dspace.ukzn.ac.za/bitstream/handle/10413/4713/Nonhlanhla%20Phakathi_MAsters%20Dissertation_08%20April%202011.pdf?sequence=1&isAllowed=y.

42. Yon Y, Mikton C, Gassoumis ZD, Wilber KH: **The prevalence of self-reported elder abuse among older women in community settings: a systematic review and meta-analysis**. *Trauma, violence & abuse* 2019, **20**(2):245-259. https://doi.org/10.1016/S2214-109X(17)30006-2.

43. Wong RT, Cafferky BM, Alejandro JP: **Chronic disease and elder mistreatment: a meta-analysis**. *International journal of geriatric psychiatry* 2021, **n/a**(n/a). https://doi.org/10.1002/gps.5640.

44. Ekot MO: **Selected demographic variables and elder abuse in Akwa Ibom state Nigeria**. *International Journal of Academic Research in Business and Social Sciences* 2016, **6**(2):1-15. Retrieved on 29th December, 2021, from: https://EconPapers.repec.org/RePEc:hur:ijarbs:v:6:y:2016:i:2:p:1-15.

45. Mouton CP, Southerland JH: **Elder abuse in the African diaspora: a review**. *Journal of Nationall Medical Association* 2017, **109**(4):262-271. https://doi.org/10.1016/j.jnma.2017.08.006.
46. Burnes D, Pillemer K, Caccamise PL, Mason A, Henderson CR, Jr., Berman J, Cook AM, Shukoff D, Brownell P, Powell M *et al*: **Prevalence of and risk factors for elder abuse and neglect in the community: a population-based study**. *Journal of the American Geriatrics Society* 2015, **63**(9):1906-1912. https://doi.org/10.1111/jgs.13601.
47. Fulmer T, Paveza G, VandeWeerd C, Fairchild S, Guadagno L, Bolton-Blatt M, Norman R: **Dyadic vulnerability and risk profiling for elder neglect**. *The Gerontologist* 2005, **45**(4):525-534. https://doi.org/10.1093/geront/45.4.525.
48. Hildebrand C, Taylor M, Bradway C: **Elder self-neglect: the failure of coping because of cognitive and functional impairments**. *Journal of the American Association of Nurse Practitioners* 2014, **26**(8):452-462. https://doi.org/10.1002/2327-6924.12045.
49. Dong X, Simon MA, Wilson RS, Mendes de Leon CF, Rajan KB, Evans DA: **Decline in cognitive function and risk of elder self-neglect: finding from the Chicago Health Aging Project**. *Journal of the American Geriatrics Society* 2010, **58**(12):2292-2299. https://doi.org/10.1111/j.1532-5415.2010.03156.x.
50. Gil AP, Kislaya I, Santos AJ, Nunes B, Nicolau R, Fernandes AA: **Elder abuse in Portugal: findings from the first national prevalence study**. *Journal of Elder Abuse & Neglect* 2015, **27**(3):174-195. https://doi.org/10.1080/08946566.2014.953659.
51. Brozowski K, Hall DR: **Aging and risk: physical and sexual abuse of elders in Canada**. *Journal of interpersonal violence* 2010, **25**(7):1183-1199. https://doi.org/10.1177/0886260509340546.
52. Nyanzi S, Nassimbwa J, Kayizzi V, Kabanda S: **'African sex is dangerous!' Renegotiating 'ritual sex' in contemporary Masaka district**. *Africa* 2008, **78**(4):518-539. https://doi.org/10.3366/E0001972008000429.
53. Ajomale, O. (2007). Elder Abuse: The Nigerian experience. *Research Committee on Sociology of Ageing (RCII).* Retrieved on 29th December, 2021, from: http://www.inpea.net/images/YinkaPaper_ElderAbuse_Nigeria_Dec07.pdf.
54. Johnson MJ, Fertel H. Elder Abuse. [Updated 2021 Aug 12]. In: StatPearls [Internet]. Treasure Island (FL): StatPearls Publishing; 2021 Jan-. Available from: https://www.ncbi.nlm.nih.gov/books/NBK560883/Jones, H., & Powell, J. L. (2006). **Old age, vulnerability and sexual violence: implications for knowledge and practice**. *International Nursing Review, 53*(3), 211-216. https://doi.org/10.1111/j.1466-7657.2006.00457.x.
55. 1. United States Census Bureau: **An Aging World: 2015**. In: *An Aging World: 2001.* Edited by Kebin K, Victoria AV, vol. 2021. US: US Department of Commerce; 2001. Retrieved on 29th December, 2021 from:: https://www.census.gov/library/publications/2001/demo/p95-01-1.html

2. World Health Organisation. (2021). Ageing. Retrieved on 29th December, 2021 from: https://www.who.int/health-topics/ageing

3. Afshar S, Roderick PJ, Kowal P, Dimitrov BD, Hill AG: **Multimorbidity and the inequalities of global ageing: A cross-sectional study of 28 countries using the World Health Surveys**. *BMC Public Health* 2015, **15**(1):776. doi:10.1186/s12889-015-2008-7

4. Center for Disease Control and Prevention. (2021). **Preventing Elder Abuse**. Retrieved on 29th December, 2021 from: https://www.cdc.gov/violenceprevention/elderabuse/fastfact.html
5. Wamara CK, Bennich M, Strandberg T: **Missing voices: older people's perspectives on being abused in Uganda**. *Journal of Elder Abuse & Neglect* 2021, **33**(4):288-310. doi:10.1080/08946566.2021.1970682
6. Atim LM, Kaggwa MM, Mamun MA, Ashaba S, Maling S: **Prevalence of severe neurocognitive impairment and its association with socio-demographics and functionality among Ugandan older persons: A hospital-based study**. *Clinical interventions in aging* 2021, **16**:1415-1425. doi:10.2147/cia.S319891
7. Kabuye R, Mukasa N: **Older people's conceptualisation of poverty and their experiences of government programmes in Uganda**. *Development in Practice* 2019, **29**(3):349-359. doi:10.1080/09614524.2018.1552659
8. Najjumba-Mulindwa, I. (2003, 2003). **Chronic poverty among the elderly in Uganda: Perceptions, experiences and policy issues.** Retrieved on 29th December, 2021 from: https://assets.publishing.service.gov.uk/media/57a08cdde5274a31e00014dc/Najjumba-Mulindwa.pdf
9. Cooper C, Livingston G: **Mental health/psychiatric issues in elder abuse and neglect**. *Clinics in geriatric medicine* 2014, **30**(4):839-850. doi:10.1016/j.cger.2014.08.011
10. World Health Organisation. (2017). **Elder abuse.** Retrieved on 29th December, 2021 from: https://www.who.int/news-room/fact-sheets/detail/elder-abuse
11. Yon Y, Mikton CR, Gassoumis ZD, Wilber KH: **Elder abuse prevalence in community settings: a systematic review and meta-analysis**. *The Lancet Global Health* 2017, **5**(2):e147-e156. https://doi.org/10.1016/S2214-109X(17)30006-2
12. Dong XQ: **Elder Abuse: Systematic Review and Implications for Practice**. *Journal of the American Geriatrics Society* 2015, **63**(6):1214-1238. doi:10.1111/jgs.13454
13. Bigala P, Ayiga N: **Prevalence and Predictors of elder abuse in Mafikeng Local Municipality in South Africa**. *Asian Population Studies* 2014, **28**:463-474. doi:10.11564/28-1-500
14. Wang XM, Brisbin S, Loo T, Straus S: **Elder abuse: an approach to identification, assessment and intervention**. *CMAJ : Canadian Medical Association journal = journal de l'Association medicale canadienne* 2015, **187**(8):575-581. doi:10.1503/cmaj.141329
15. National Research Council Panel to Review, Risk and Prevalence of Elder Abuse and Neglect: **The National Academies Collection: Reports funded by National Institutes of Health**. In: *Elder Mistreatment: Abuse, Neglect, and Exploitation in an Aging America.* edn. Edited by Bonnie RJ, Wallace RB. Washington (DC): National Academies Press (US) Copyright © 2003, National Academy of Sciences.; 2003.
16. Storey JE: **Risk factors for elder abuse and neglect: A review of the literature**. *Aggression and Violent Behavior* 2020, **50**:101339. https://doi.org/10.1016/j.avb.2019.101339
17. Dong X, Chen R, Chang ES, Simon M: **Elder abuse and psychological well-being: A systematic review and implications for research and policy - a mini review**. *Gerontology* 2013, **59**(2):132-142. doi:10.1159/000341652

18. Baker MW, Sugar NF, Eckert LO: **Sexual assault of older women: Risk and vulnerability by living arrangement**. *Sexuality Research and Social Policy Journal of NSRC* 2009, **6**(4):79-87. doi:10.1525/srsp.2009.6.4.79

19. Lachs MS, Pillemer K: **Abuse and neglect of elderly persons**. *The New England journal of medicine* 1995, **332**(7):437-443. doi:10.1056/nejm199502163320706

20. Johnson MJ, Fertel H. Elder Abuse. [Updated 2021 Aug 12]. In: StatPearls [Internet]. Treasure Island (FL): StatPearls Publishing; 2021 Jan-. Available from: https://www.ncbi.nlm.nih.gov/books/NBK560883/Jones, H., & Powell, J. L. (2006). Old age, vulnerability and sexual violence: Implications for knowledge and practice. *International Nursing Review, 53*(3), 211-216. doi:https://doi.org/10.1111/j.1466-7657.2006.00457.x.

21. Neale AV, Hwalek MA, Scott RO, Sengstock MC, Stahl C: **Validation of the Hwalek-Sengstock Elder Abuse Screening Test**. *Journal of Applied Gerontology* 1991, **10**(4):406-418. doi:10.1177/073346489101000403

22. Ozmete E: **The Hwalek-Sengstock Elder Abuse Screening Test: The adaptation study into Turkish/Hwalek-Sengstock Yasli Istismari Tarama Testi: Turkceye uyarlama calismasi**. *Anadolu Psikiyatri Dergisi* 2016, **17**(S1):45-53.

23. Aminalroaya R, Alizadeh-Khoei M, Hormozi S, Sharifi F, Taati F: **Screening for elder abuse in geriatric outpatients: reliability and validity of the Iranian version Hwalek-Sengstock Elder Abuse Screening Test (H-S/EAST)**. *J Elder Abuse & Neglect* 2020, **32**(1):84-96. doi:10.1080/08946566.2020.1719564

24. Mahoney FI, Barthel DW: **Functional evaluation: The Barthel Index**. *Maryland state medical journal* 1965, **14**:61-65.

25. Saikia, A. M., Mahanta, N., Mahanta, A., Deka, A. J., & Kakati, A. (2015). **Prevalence and risk factors of abuse among community dwelling elderly of Guwahati City, Assam.** *Indian journal of community medicine : official publication of Indian Association of Preventive & Social Medicine, 40*(4), 279-281. doi:10.4103/0970-0218.164406.

26. Fulmer T, Strauss S, Russell SL, Singh G, Blankenship J, Vemula R, Caceres B, Valenti M, Sutin D: **Screening for elder mistreatment in dental and medical clinics**. *Gerodontology* 2012, **29**(2):96-105. doi:10.1111/j.1741-2358.2010.00405.x

27. Wu L, Chen H, Hu Y, Xiang H, Yu X, Zhang T, Cao Z, Wang Y: **Prevalence and associated factors of elder mistreatment in a rural community in People's Republic of China: a cross-sectional study**. *PloS one* 2012, **7**(3):e33857-e33857. doi:10.1371/journal.pone.0033857

28. Ramalingam A, Sarkar S, Premarajan KC, Rajkumar RP, Subrahmanyam DK: **Prevalence and correlates of elder abuse: A cross-sectional, community-based study from rural Puducherry**. *The National medical journal of India* 2019, **32**(2):72-76. doi:10.4103/0970-258x.275344

29. Hosseinkhani Z, Moradi Z, Khodamoradi F: **Elder abuse: Screening in Iranian families**. *Medical journal of the Islamic Republic of Iran* 2017, **31**:126. doi:10.14196/mjiri.31.126

30. Rosen T, Mehta-Naik N, Elman A, Mulcare MR, Stern ME, Clark S, Sharma R, LoFaso VM, Breckman R, Lachs M *et al*: **Improving Quality of Care in Hospitals for Victims of Elder Mistreatment: Development of the Vulnerable Elder Protection

Team. *Joint Commission journal on quality and patient safety* 2018, **44**(3):164-171. doi:10.1016/j.jcjq.2017.08.010

31. Skirbekk V, James KS: **Abuse against elderly in India – The role of education**. *BMC Public Health* 2014, **14**(1):336. doi:10.1186/1471-2458-14-336
32. Sathya T, Premkumar R: **Association of functional limitations and disability with elder abuse in India: a cross-sectional study**. *BMC Geriatrics* 2020, **20**(1):220. doi:10.1186/s12877-020-01619-3
33. Cadmus EO, Owoaje ET: **Prevalence and correlates of elder abuse among older women in rural and urban communities in South Western Nigeria**. *Health care for women international* 2012, **33**(10):973-984. doi:10.1080/07399332.2012.655394
34. El-Khawaga G, Eladawi N, Abdel-Wahab F: **Abuse of Rural Elders in Mansoura Districts, Dakahlia, Egypt: Prevalence, Types, Risk Factors, and Lifestyle**. *Journal of interpersonal violence* 2021, **36**(5-6):Np2868-np2882. doi:10.1177/0886260518767900
35. Simona , V., & Statista. (2021). **GDP per capita of African countries 2021**. Retrieved on 29th December, 2021 from: https://www.statista.com/statistics/1120999/gdp-of-african-countries-by-country/
36. Wood S, Lichtenberg PA: **Financial capacity and financial exploitation of older adults: Research findings, policy recommendations and clinical implications**. *Clinical Gerontology* 2017, **40**(1):3-13. doi:10.1080/07317115.2016.1203382
37. Kohn R, Verhoek-Oftedahl W: **Caregiving and elder abuse**. *Medicine and health, Rhode Island* 2011, **94**(2):47-49. https://pubmed.ncbi.nlm.nih.gov/21456376/
38. Pillemer K, Burnes D, Riffin C, Lachs MS: **Elder Abuse: Global Situation, Risk Factors, and Prevention Strategies**. *The Gerontologist* 2016, **56 Suppl 2**(Suppl 2):S194-205. doi:10.1093/geront/gnw004
39. Akpan ID, Umobong ME: **An Assessment of the Prevalence of Elder Abuse and Neglect in Akwa Ibom State, Nigeria**. *Developing Country Studies* 2013, **3**:8-14. Retrieved on 29th December, 2021 from: https://www.iiste.org/Journals/index.php/DCS/article/view/5493
40. Phakathi NN: **Exploring elder abuse among clinic attendees in a selected Durban hospital**. *Masters dissertation.* South Africa: University of KwaZulu-Natal; 2011. Retrieved on 29th December, 2021 from: https://ukzn-dspace.ukzn.ac.za/bitstream/handle/10413/4713/Nonhlanhla%20Phakathi_MAsters%20Dissertation_08%20April%202011.pdf?sequence=1&isAllowed=y
41. Yon Y, Mikton C, Gassoumis ZD, Wilber KH: **The Prevalence of Self-Reported Elder Abuse Among Older Women in Community Settings: A Systematic Review and Meta-Analysis**. *Trauma, violence & abuse* 2019, **20**(2):245-259. https://doi.org/10.1016/S2214-109X(17)30006-2
42. Wong RT, Cafferky BM, Alejandro JP: **Chronic disease and elder mistreatment: A meta-analysis**. *International journal of geriatric psychiatry* 2021, **n/a**(n/a). https://doi.org/10.1002/gps.5640
43. Ekot MO: **Selected Demographic Variables and Elder Abuse in Akwa Ibom State Nigeria**. *International Journal of Academic Research in Business and Social Sciences* 2016, **6**(2):1-15. Retrieved on 29th December, 2021 from: https://EconPapers.repec.org/RePEc:hur:ijarbs:v:6:y:2016:i:2:p:1-15

44. Mouton CP, Southerland JH: **Elder Abuse in the African Diaspora: A Review**. *J Natl Med Assoc* 2017, **109**(4):262-271. https://doi.org/10.1016/j.jnma.2017.08.006

45. Burnes D, Pillemer K, Caccamise PL, Mason A, Henderson CR, Jr., Berman J, Cook AM, Shukoff D, Brownell P, Powell M *et al*: **Prevalence of and Risk Factors for Elder Abuse and Neglect in the Community: A Population-Based Study**. *Journal of the American Geriatrics Society* 2015, **63**(9):1906-1912. doi:10.1111/jgs.13601

46. Fulmer T, Paveza G, VandeWeerd C, Fairchild S, Guadagno L, Bolton-Blatt M, Norman R: **Dyadic vulnerability and risk profiling for elder neglect**. *The Gerontologist* 2005, **45**(4):525-534. doi:10.1093/geront/45.4.525

47. Hildebrand C, Taylor M, Bradway C: **Elder self-neglect: the failure of coping because of cognitive and functional impairments**. *Journal of the American Association of Nurse Practitioners* 2014, **26**(8):452-462. doi:10.1002/2327-6924.12045

48. Dong X, Simon MA, Wilson RS, Mendes de Leon CF, Rajan KB, Evans DA: **Decline in cognitive function and risk of elder self-neglect: finding from the Chicago Health Aging Project**. *Journal of the American Geriatrics Society* 2010, **58**(12):2292-2299. doi:10.1111/j.1532-5415.2010.03156.x

49. Gil AP, Kislaya I, Santos AJ, Nunes B, Nicolau R, Fernandes AA: **Elder abuse in Portugal: findings from the first national prevalence study**. *Journal of Elder Abuse & Neglect* 2015, **27**(3):174-195. doi:10.1080/08946566.2014.953659

50. Brozowski K, Hall DR: **Aging and risk: physical and sexual abuse of elders in Canada**. *Journal of interpersonal violence* 2010, **25**(7):1183-1199. doi:10.1177/0886260509340546

51. Nyanzi S, Nassimbwa J, Kayizzi V, Kabanda S: **'African Sex is Dangerous!' Renegotiating 'Ritual Sex' in Contemporary Masaka District**. *Africa* 2008, **78**(4):518-539. doi:10.3366/E0001972008000429

52. Ajomale, O. (2007). Elder Abuse: The Nigerian experience. *Research Committee on Sociology of Ageing (RCII).* Retrieved on 29th December, 2021 from: http://www.inpea.net/images/YinkaPaper_ElderAbuse_Nigeria_Dec07.pdf

53. Johnson MJ, Fertel H. Elder Abuse. [Updated 2021 Aug 12]. In: StatPearls [Internet]. Treasure Island (FL): StatPearls Publishing; 2021 Jan-. Available from: https://www.ncbi.nlm.nih.gov/books/NBK560883/Jones, H., & Powell, J. L. (2006). **Old age, vulnerability and sexual violence: Implications for knowledge and practice**. *International Nursing Review, 53*(3), 211-216. doi:https://doi.org/10.1111/j.1466-7657.2006.00457.x

54. Cybulska B: **Sexual assault: key issues**. *Journal of the Royal Society of Medicine* 2007, **100**(7):321-324. https://doi.org/10.1177/014107680710000713.

Table 1. Characteristics of the Participants

**Variables**	**n (%)**
**Age**	
60 – 69	252 (69.0)
70 – 79	90 (24.7)
Above 80	23 (6.3)
**Gender**	
Female	208 (57.0)
Male	157 (43.0)
**Area of dwelling**	
Rural	259 (71.0)
Urban	106 (29.0)
**Marital status**	
Cohabiting or married	217 (59.4)
Divorced or separated	58 (15.9)
Never married	3 (0.8)
Widowed	87 (23.8)
**Employment status**	
Previously formally employed, retired but currently still active	24 (6.6)
Previously formally employed, retired but currently not active	41 (11.23)
Previously informally employed, currently not active	43 (11.8)
Previously informally employed and currently still active	257 (70.4)
Level of education	
Never	86 (23.6)
Primary	165 (45.2)
Secondary	70 (19.2)
Post-secondary	44 (12.0)
**Type of housing**	
Private	325 (89.0)
Public	16 (4.4)
Rental	24 (6.6)
**Presence of a chronic illness**	
No	101 (27.7)
Yes	264 (72.3)

**Physical impairment**	
No	203 (55.6)
Yes	162 (44.4)
**Reported perpetrators**	
No	299 (81.9)
Yes	66 (18.1)
**History of reporting abuse to police**	
Declines	332 (91.0)
Reported	33 (9.0)
**Functional dependence**	
Severe dependency	11
Moderate dependency	69
Slight dependency	48
Independent	237

Table 2. Associations between elderly abuse severity and the studied variables

**Variables**	**Elderly abuse severity**		**Bivariate linear regression**		**Multivariate linear regression**	
	Mean (SD)	F(p-value)	coefficient (standard beta)	p-value	coefficient (standard beta)	p-value
**Age**						
60 – 69	6.02 (2.74)	0.84 (0.614)	1			
70 – 79	6.09 (2.57)		0.07 (0.01)	0.834		
Above 80	5.70 (2.30)		-0.32 (-0.03)	0.578		
**Gender**						
Female	6.23 (2.66)	1.73 (0.084)	1			
Male	5.74 (2.67)		-0.49 (-0.09)	0.084		
**Area of dwelling**						
Rural	5.99 (2.70)	-0.27 (0.797)	1			
Urban	6.07 (2.59)		0.08 (0.01)	0.787		
**Marital status**						
Cohabiting or married	5.79 (2.57)	2.20 (0.088)	1		1	
Divorced or separated	6.79 (2.38)		1.00 (0.14)	0.011	0.74 (0.10)	0.061
Never married	5.67 (2.89)		-0.13 (-0.01)	0.935	-0.95 (-0.03)	0.538
Widowed	6.07 (2.58)		0.28 (0.04)	0.413	0.26 (0.04)	0.434
**Employment status**						

Previously formally employed, retired but currently still active	4.96 (2.53)	2.06 (0.105)	1		1	
Previously formally employed, retired but currently not active	5.71 (3.11)		0.75 (0.09)	0.273	0.35 (0.04)	0.603
Previously informally employed, currently not active	5.74 (2.29)		0.79 (0.09)	0.246	0.16 (0.02)	0.823
Previously informally employed and currently still active	6.21 (2.65)		1.25 (0.21)	0.028	0.59 (0.10)	0.363
**Level of education**						
Never	5.64 (2.63)	5.03 (0.002)	1		1	
Primary	6.21 (2.69)		0.57 (0.11)	0.106	0.63 (0.12)	0.072
Secondary	6.71 (2.54)		1.07 (0.16)	0.011	0.97 (0.14)	0.028
Post-secondary	4.93 (2.50)		-0.71 (-0.09)	0.147	-0.31 (-0.04)	0.606
**Type of housing**						
Private	5.90 (2.67)	4.15 (0.016)	1		1	
Public	7.81 (2.10)		1.91 (0.15)	0.005	1.08 (0.08)	0.121
Rental	6.33 (2.66)		0.43 (0.04)	0.444	0.40 (0.04)	0.476
**Presence of a chronic illness**						
No	5.98 (2.71)	-0.16 (0.873)	1			
Yes	6.03 (2.66)		0.05 (0.01)	0.873		
**Physical impairment**						
No	5.56 (2.72)	-3.71 (<0.001)	1		1	
Yes	6.59 (2.50)		1.02 (0.19)	<0.001	0.83 (0.15)	0.003
**Reported perpetrators**						
No	5.90 (2.61)	-1.84 (0.067)	1			
Yes	6.56 (2.89)		0.66 (0.96)	0.067		
**History of reporting abuse to police**						
Declines	5.93 (2.63)		1			
Reported	6.88 (2.92)	-1.95 (0.051)	0.95 (0.10)	0.051		
**Functional dependence**						
Severe dependency	5.18 (2.75)	0.62 (0.600)	1			
Moderate dependency	5.97 (2.29)		0.79 (0.11)	0.364		
Slight dependency	6.35 (2.85)		1.17 (0.15)	0.190		

Independent	6.00 (2.73)		0.82 (0.15)	0.322		

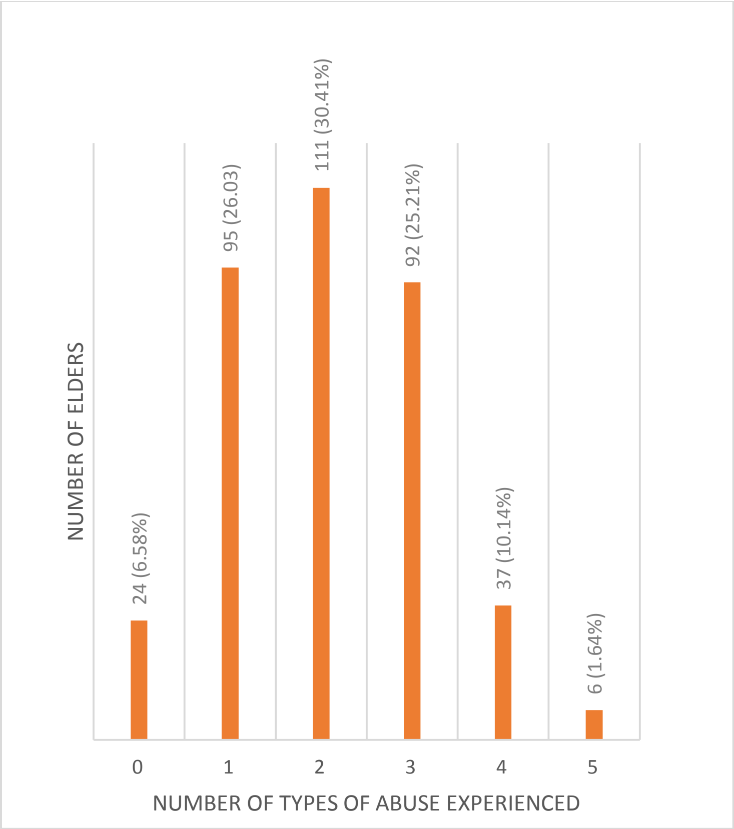

Figure 1: Histogram of number of abuses experienced by the elders

Table 3: Participants experiencing two types of abuse

**Type of abuse**	1	2	3	4	5
Sexual (1)	**-**				
Emotional (2)	**8**	**-**			
Physical mistreatment (3)	**14**	**113**	**-**		
Neglect (4)	**5**	**15**	**243**	**-**	
Financial (5)	**11**	**83**	**28**	**159**	**-**

Table 4 Correlation between the different types of abuse and elderly abuse severity

**Type of abuse**	1	2	3	4	5	6
Sexual (1)	1					
Emotional (2)	**0.10***	1				
Physical mistreatment (3)	**0.14****	**0.35****	1			
Neglect (4)	-0.05	**0.15****	0.04	1		
Financial (5)	0.05	**0.13***	**0.20****	**0.13****	1	
Elderly abuse severity (6)	0.03	**0.17****	**0.17****	-0.11	-0.48	1
* = *p* value less than 0.05; ** = *p* value less than 0.001; very high correlation positive (negative) = r^2 0.90 to 1.00 (−0.90 to −1.00); high positive (negative) correlation = 0.70 to 0.90 (−0.70 to −0.90); moderate positive (negative) correlation = 0.50 to 0.70 (−0.50 to −0.70); low positive (negative) correlation = 0.30 to 0.50 (−.30 to −0.50); Negligible correlation = 0.00 to 0.30 (.00 to −0.30).						

Table 5: Multivariable logistic regression analysis for factors associated with the different types of abuse

Variables	Neglect		Financial abuse		Emotional mistreatment		Physical mistreatment	
	aOR (95% CI)	p-value	aOR (95% CI)	p-value	aOR (95% CI)	p-value	aOR (95% CI)	p-value
Age								
60 – 69			1				1	
70 – 79			1.15 (0.67 – 1.99)	0.606			0.71 (0.35 – 1.43)	0.337
Above 80			11.24 (2.37 – 53.21)	0.002			1.83 (0.68 – 4.88)	0.227
Gender								
Female			1					
Male			1.25 (0.77 – 2.03)	0.367				
Area of dwelling								
Rural			1					
Urban			1.37 (0.80 – 2.35)	0.249				
Level of education								
Never			1					
Primary			2..01 (1.08 – 3.72)	0.027				
Secondary			1.27 (0.61 – 2.66)	0.523				
Tertiary			3.65 (1.48 – 9.02)	0.005				
Type of housing								
Private	1							
Public	0.60 (0.18 – 1.98)	0.397						
Rental	0.83 (0.22 – 3.15)	0.783						
Presence of a chronic illness								
No			1		1		1	
Yes			0.75 (0.43 – 1.29)	0.296	0.61 (0.37 – 1.01)	0.053	0.40 (0.23 – 0.71)	0.002
Physical impairment								
No	1		1					
Yes	0.27 (0.14 – 0.52)	<0.001	0.40 (0.24 – 0.65)	<0.001				
Reported perpetrators								
No	1				1		1	
Yes	3.49 (1.01 – 12.03)	0.047			7.94 (3.71 – 17.06)	<0.001	5.14 (2.79 – 9.44)	<0.001
History of reporting abuse to police								
No			1		1			
Yes			0.31 (0.12 – 0.75)	0.009	3.33 (1.25 – 8.86)	0.016		
Functional dependence								
Severe dependency	Omitted		8.95 (1.57 – 51.08)	0.014	0.52 (0.12 – 2.22)	0.533	0.32 (0.04 – 2.89)	0.312
Moderate dependency	9.33 (2.15 – 40.42)	0.003	1.73 (0.92 – 3.26)	0.090	1.85 (1.01 – 3.78)	0.047	2.44 (1.13 – 4.08)	0.019
Slight dependency	6.48 (1.48 – 28.34)	0.013	1.01 (0.50 – 2.03)	0.980	1.04 (0.53 – 2.05)	0.898	0.78 (0.31 – 1.95)	0.597

Independent	1		1		1		1	

Supplemetary file 1

Click here to access/download
Supporting Information
Supplementary File 1.docx

Supplemetary file 2

Click here to access/download
Supporting Information
Supplementary file 2.docx

Supplemetary file 3

Click here to access/download
Supporting Information
Supplemntary file 3.docx

Click here to access/download
Supporting Information - Compressed/ZIP File Archive
Supplementary file 4.rar
